# Screening for Active Compounds Targeting Human Natural Killer Cell Activation Identifying Daphnetin as an Enhancer for IFN-γ Production and Direct Cytotoxicity

**DOI:** 10.3389/fimmu.2021.680611

**Published:** 2021-12-08

**Authors:** Baige Yao, Qinglan Yang, Yao Yang, Yana Li, Hongyan Peng, Shuting Wu, Lili Wang, Shuju Zhang, Minghui Huang, Erqiang Wang, Peiwen Xiong, Ting Luo, Liping Li, Sujie Jia, Yafei Deng, Youcai Deng

**Affiliations:** ^1^ Hunan Children’s Research Institute (HCRI), Hunan Children’s Hospital, Changsha, China; ^2^ Department of Pharmacy, The Third Xiangya Hospital, Central South University, Changsha, China; ^3^ Hunan Provincial Key Laboratory of Children’s Emergency Medicine, Hunan Children’s Hospital, Changsha, China; ^4^ Institute of Materia Medica, College of Pharmacy, Army Medical University (Third Military Medical University), Chongqing, China

**Keywords:** natural killer cell, Daphnetin, interferon (IFN)-γ, PI3K-Akt, mTOR

## Abstract

Natural killer (NK) cells are a potent weapon against tumor and viral infection. Finding active compounds with the capacity of enhancing NK cell effector functions will be effective to develop new anti-cancer drugs. In this study, we initially screened 287 commercially available active compounds by co-culturing with peripheral blood mononuclear cells (PBMCs). We found that five compounds, namely, Daphnetin, MK-8617, LW6, JIB-04, and IOX1, increased the IFN-γ^+^ NK cell ratio in the presence of IL-12. Further studies using purified human primary NK cells revealed that Daphnetin directly promoted NK cell IFN-γ production in the presence of IL-12 but not IL-15, while the other four compounds acted on NK cells indirectly. Daphnetin also improved the direct cytotoxicity of NK cells against tumor cells in the presence of IL-12. Through RNA-sequencing, we found that PI3K-Akt-mTOR signaling acted as a central pathway in Daphnetin-mediated NK cell activation in the presence of IL-12. This was further confirmed by the finding that both inhibitors of PI3K-Akt and its main downstream signaling mTOR, LY294002, and rapamycin, respectively, can reverse the increase of IFN-γ production and cytotoxicity in NK cells promoted by Daphnetin. Collectively, we identify a natural product, Daphnetin, with the capacity of promoting human NK cell activation *via* PI3K-Akt-mTOR signaling in the presence of IL-12. Our current study opens up a new potential application for Daphnetin as a complementary immunomodulator for cancer treatments.

## Introduction

Natural killer (NK) cells, which are an important member of the innate immune system, play a pivotal role in immunosurveillance against tumor cells and viral infection (1). NK cells can directly kill infected, foreign, and transformed cells by secreting perforin and granzyme B, and also regulate various immune responses by secreting several cytokines, including interferon gamma (IFN-γ) (2–4). These properties make NK cells a potent weapon against cancers. Several strategies of NK cell-based immunotherapy have been applied in the clinic, including adoptive transfer of NK cells, direct stimulation, blockade of NK cell inhibitory receptors, recruitment of NK cells into the tumor microenvironment, and modulation of the tumor microenvironment (4, 5). A recent study has demonstrated that adoptive transfer of allogeneic NK cells for cancer treatment shows good therapeutic effects without obvious fatal side effects, such as cytokine release storm and graft *versus* host diseases (6), which draws great attention in the field of cancer immunotherapy.

IFN-γ is a type II interferon that plays an important role in both host defense and immune regulation. Recombinant IFN-γ has been used in the clinic as an anti-cancer reagent (7). As one of the major sources of IFN-γ, NK cell-derived IFN-γ mediates T helper 1 cell differentiation (8) and also promotes differentiation of CD8^+^ T cells into cytotoxic effector cells (9). Meanwhile, IFN-γ is a potent macrophage activator by initiating nitric oxide production, upregulating co-stimulatory molecule expression and increasing phagocytic function (8). In our previous studies, we screened several natural products that could enhance NK cell effector function by initially determining IFN-γ production. We reported two natural products, phyllanthusmin C (PL-C) and 4-O-{[2’’,3’’,4’’-tri-O-acetyl-α-D-arabinopyranosyl-(1’’→4’)]-2’,3’-di-O-acetyl-α-L-rhamnopyranosyl} diphyllin (TAARD), that could promote NK cell-derived IFN-γ production *via* the NF-кB signal pathway (10, 11). Therefore, screening of molecules that can enhance NK cell IFN-γ production will be effective to find new candidates to improve NK cell effector function for cancer immunotherapy (12, 13).

IL-12 and IL-15, mainly produced by antigen-processing cells when encountering transformed cells, are critical for NK cell activation and have been used in several clinical trials, including melanoma, glioma, and metastatic non-small cell lung cancer. However, high dose usage of these cytokines usually causes toxicity or pleiotropic effects (14). Thus, reducing the dose of IL-12 or IL-15 or expressing them at the site of tumor is an alternative way to attenuate their systemic side effects.

In this study, we screened 287 active compounds that could be acquired commercially by initially determining their capacity in regulating IFN-γ production by NK cells in the presence of IL-12. We successfully identified a dihydroxylated derivative of coumarin, Daphnetin, which could enhance both the IFN-γ production and cytotoxicity of NK cells *via* regulating the PI3K-Akt-mTOR signal pathway in the presence of IL-12.

## Materials and Methods

### Active Compound Library

The active compound library, which consists of 287 compounds, including histone deacetylase inhibitor (HDACi), sirtuin inhibitor (Sirti), DNA methyltransferase inhibitor (DNMTi), histone Methyl Transferase inhibitor (HMTi), and histone acetyltransferase inhibitor (HATi), was purchased from Selleckchem (https://www.selleckchem.com/screening/epigenetics-compound-library.html) (Houston, TX, United States) and stored at −80°C. A total of 267 compounds were pre-dissolved at 10 mM in dimethyl sulfoxide (DMSO), 9 compounds were pre-dissolved at 2 mM in DMSO, and 11 compounds were pre-dissolved at 10 mM in H_2_O.

### Isolation of Peripheral Blood Mononuclear Cells and NK Cells

Peripheral blood from healthy donors (age range from 18 to 55 years) was obtained from Changsha Blood Center (Changsha, China), with authorization number HCHLL-2019-48 from the Institutional Review Board of Hunan Children’s Hospital. PBMCs were isolated from leucocytes *via* Ficoll-Hypaque density gradient centrifugation (GE Healthcare Bio-Sciences, Pittsburgh, PA, United States), as described previously (11). NK cells were enriched *via* negative selection using a human NK cell isolation kit (Miltenyi Biotec, San Diego, CA, United States), followed by cell sorting (FACS Aria III cell sorter, BD Biosciences, San Jose, CA, United States). NK cells were sorted as CD56-positive and CD3-negative (BioLegend, San Diego, CA, United States), as described previously (11). The purity of CD56^+^CD3^-^ NK cells was ≥99.0%.

### Cell Culture and Treatment

K562 cells were commercially purchased with authentication from the National Collection of Authenticated Cell Cultures of China. K562 cells had been tested and confirmed negative for mycoplasma contamination by using a Mycoplasma PCR Detection Kit (Sigma-Aldrich, St. Louis, MO, USA). K562 cells, PBMCs, and purified human primary NK cells mentioned later were cultured in RPMI 1640 medium (Gibco, Grand Island, NY, USA) supplemented with 10% heat-inactivated fetal bovine serum (Biological Industries, Kibbutz Beit Haemek, Israel), 1% penicillin, and 1% streptomycin at 37°C in 5% CO_2_.

PBMCs at a density of 5 × 10^6^ cells/ml or purified human primary NK cells at a density of 1 × 10^6^ cells/ml were resuspended and seeded into a 96-well plate with 200 μl of medium per well. After resting for 2 h, cells were treated with DMSO or 10 μM indicated active compounds in the presence of IL-12 (10 ng/ml), IL-15 (10 ng/ml), or IL-12 (10 ng/ml) plus IL-18 (10 ng/ml) (BioLegend) for 18 h, as indicated. Then, the cell culture supernatants were collected for determination of IFN-γ protein levels by ELISA with commercially available mAb pairs according to the manufacturer’s protocol (BioLegend), while cell pellets were harvested for protein extraction to perform immunoblotting. If cells were harvested for flow cytometric analysis of IFN-γ production in NK cells, 1 µl/ml Golgi Plug and Golgi Stop (BD Biosciences) was added 5 h before cells were harvested.

For PBMCs or purified human primary NK cells and K562 cell co-culture, 2.5 × 10^6^ PBMCs or purified human primary NK cells were first activated with indicated compounds in the presence of IL-12 (10 ng/ml) in a 96-well V-bottom plate for 18 h, followed by adding K562 cells for another 6 h. Then, the cell culture supernatants were collected for determination of IFN-γ protein levels by ELISA.

For the PI3K-Akt-mTOR inhibitor assay, PBMCs or purified human primary NK cells were treated with or without LY294002 (10 μM) or rapamycin (1 nM) in the presence of IL-12 (10 ng/ml) and Daphnetin for 60 min or 18 h. Then, the supernatants of cell culture were collected for determination of IFN-γ protein levels by ELISA and cell pellets were harvested for protein extraction to perform immunoblotting. If cells were harvested for flow cytometric analysis of IFN-γ production in NK cells, 1 µl/ml Golgi Plug and Golgi Stop were added 5 h before cells were harvested.

### Flow Cytometric Analysis

Flow cytometric analysis was performed as described previously (15, 16), and all antibodies are listed in **Supplementary Table 1**. For surface staining of human natural cytotoxicity triggering receptor 1 (NCR1, also called as NKp46), NCR3 (also called as NKp30), NCR2 (also called as NKp44), killer cell lectin-like receptor K1 (KLRK1, also called as NKG2D), CD244 (also called as 2B4), Fc fragment of IgG receptor IIIa (also called as CD16), TNF superfamily member 10 (TNFSF10, also called as TRAIL), CD226 (also called as DNAM-1), killer cell lectin like receptor C1 (KLRC1, also called as NKG2A), and factor associated suicide ligand (FasL), NK cells were stained with indicated antibodies at room temperature for 15 min in the dark with staining buffer [phosphate-buffered saline (PBS) containing 0.5% fetal bovine serum]. For intracellular staining of Ki67, purified human primary NK cells were harvested and resuspended in Foxp3/Transcription Factor staining buffer (eBioscience, San Diego, CA, USA) at 4°C for 2 h. After fixing and permeabilizing, cells were stained with anti-Ki67-FITC antibody at room temperature for 1 h. For apoptosis staining, cells were harvested and stained with AnnexinV-FITC and 7-AAD antibodies at room temperature for 15 min in the dark with 1× binding buffer. For intracellular staining of IFN-γ, cells were stained with human CD3-PE-Cy7 and CD56-APC antibodies at room temperature for 15 min. Then, cells were washed and resuspended in Cytofix/Cytoperm solution (BD Biosciences) at 4°C for 20 min. After fixing and permeabilizing, cells were stained with anti-IFN-γ-FITC antibodies for 30 min. Labeled cells were detected using LSRFortessa Flow Cytometer (BD Biosciences) and data were further analyzed by FlowJo 10.5.3 software (Tree Star, Ashland, OR).

### ELISA of IFN-γ Secretion in Cell Culture Supernatants

The concentration of IFN-γ in the supernatants of cell culture was measured by ELISA kits according to the manufacturer’s instructions. The absorbance at 570 nm can be subtracted from the absorbance at 450 nm.

### CD107a Degranulation Assay

CD107a degranulation assay was performed as described previously (17). Briefly, PMBCs or purified human primary NK cells were initially activated with DMSO or Daphnetin in the presence or absence of inhibitors for 18 h before K562 cell co-culture. Then, the pre-activated cells were cultured with K562 cells at a 5:1 ratio in the presence of the anti-CD107a-FITC antibodies (BD Biosciences) and protein transport inhibitor (Golgi Plug and Golgi Stop). After 6 h of co-culture at 37°C, the ratio of CD107a^+^ NK cells was determined by flow cytometry.

### NK Cells Cytotoxicity Assay

For K562 cell apoptosis assay, purified human primary NK cells were initially activated with DMSO or Daphnetin for 18 h in 96-well V-plate. The K562 cells were labeled with CTV dye (ThermoFisher Scientific, Waltham, United States) and then added to the pre-activated NK cells for co-culture at a 5:1 ratio for another 6 h. The cells were harvested and then the apoptosis kit was used and the ratio of Annexin V^-^7-AAD^-^, Annexin V^+^7-AAD^-^, and Annexin V^+^7-AAD^+^ K562 cells was determined by flow cytometry (18).

NK cell cytotoxicity assay against K562 cells was also performed by using a real-time digital bio-imaging system, as described previously (19). Briefly, purified human primary NK cells were initially activated with DMSO or Daphnetin in the presence of IL-12 for 18 h in a 96-well U plate. The K562 cells were labeled with CellTracker dye (ThermoFisher Scientific, Waltham, United States) and then added to the pre-activated NK cells for co-culture at different ratios. Next, the plate was placed in a cell-imaging multimode reader (Cytation 5, Biotek, VT, United States) and the protocol was set to focus on CellTracker-labeled K562 cells in each well at the indicated time points. The fluorescent area of CellTracker-labeled K562 cells in each well was recorded at 4 and 8 h and further analyzed using Gen5™ software (BioTek, VT, United States).

### RNA-Sequencing and Bioinformatics Analysis

Purified human primary NK cells were treated with DMSO or Daphnetin in the presence of IL-12 (10 ng/ml) for 12 h. After that, the cells were collected and total RNA was extracted using the Total RNA purification Micro Kit (Norgen biotek, Thorold, Canada). The quantification of total RNA was assessed using Qubit 4.0 (Invitrogen, Waltham, MA, USA). The cDNA library preparation was performed using the Illumina TruSeq RNA sample preparation kit and the qualities were assessed using an Agilent 2100 Bioanalyzer with Agilent High Sensitivity DNA Kit (Agilent Technologies, Palo Alto, CA). For sequencing, the cDNA libraries were loaded on an Illumina HiSeq 2500 at Biomarker Technologies Co. Ltd (Beijing, China). The raw sequence reads in fastq format were processed and analyzed. Briefly, the sequencing quality was first assessed using FastQC, and poor-quality 5′end reads were trimmed using a custom Perl script. HISAT2 was used for the probabilistic alignment of reads to the reference genome index (hg38) with default parameters (20). StringTie was used to perform transcript-based quantification and estimated transcripts based on the reference annotation file (21). Limma/voom was used for identifying differentially expressed genes (DEGs) from paired samples based on raw read counts. Significantly, DEGs were defined as those with an adjusted *p*-value < 0.05, and fold change > 1.5 (22). R package cluster Profiler was used for the Kyoto Encyclopedia of Genes and Genomes (KEGG) pathway enrichment analysis of significant DEGs, and *q*-value < 0.05 was chosen to report (23, 24). Gene Set Enrichment Analysis (GSEA) was performed with indicated prognostic by clusterProfiler and enrichplot (24). Protein–protein interaction (PPI) network was constructed by String (https://string-db.org), and Cytoscpe V3.6.0 was used to show the network (25).

### Immunoblotting

After being collected, the cell pellets were washed with iced PBS and then directly lysed in 2 × SDS-PAGE sample loading buffer supplemented with 2.5% β-mercaptoethanol, denatured at 100°C for 8 min and then subjected to immunoblotting analysis as described previously (11). Antibodies against p-p85, p-Akt^ser473^, p-Akt^thr308^, p-S6^ser235/236^, p-STAT3, p-STAT4, p-STAT5, and β-actin (Cell Signaling Technology, Danvers, MA) were used for immunoblotting (listed in **Supplementary Table 1**). β-actin protein was used as the internal reference.

### Statistical Analysis

All data were analyzed using GraphPad Prism 8.0 for Windows software (GraphPad Software Inc, La Jolla, CA, United States). Pairwise comparisons were conducted by paired *t*-test from the same donor. In each analysis, results were representative of at least three independent experiments and three to eight replicates per experiment. *p-*value < 0.05 or less was considered statistically significant.

## Results

### Active Compound Screening of Human Primary NK Cell Activator *via* Determining IFN-γ Production

**Figure 1:**
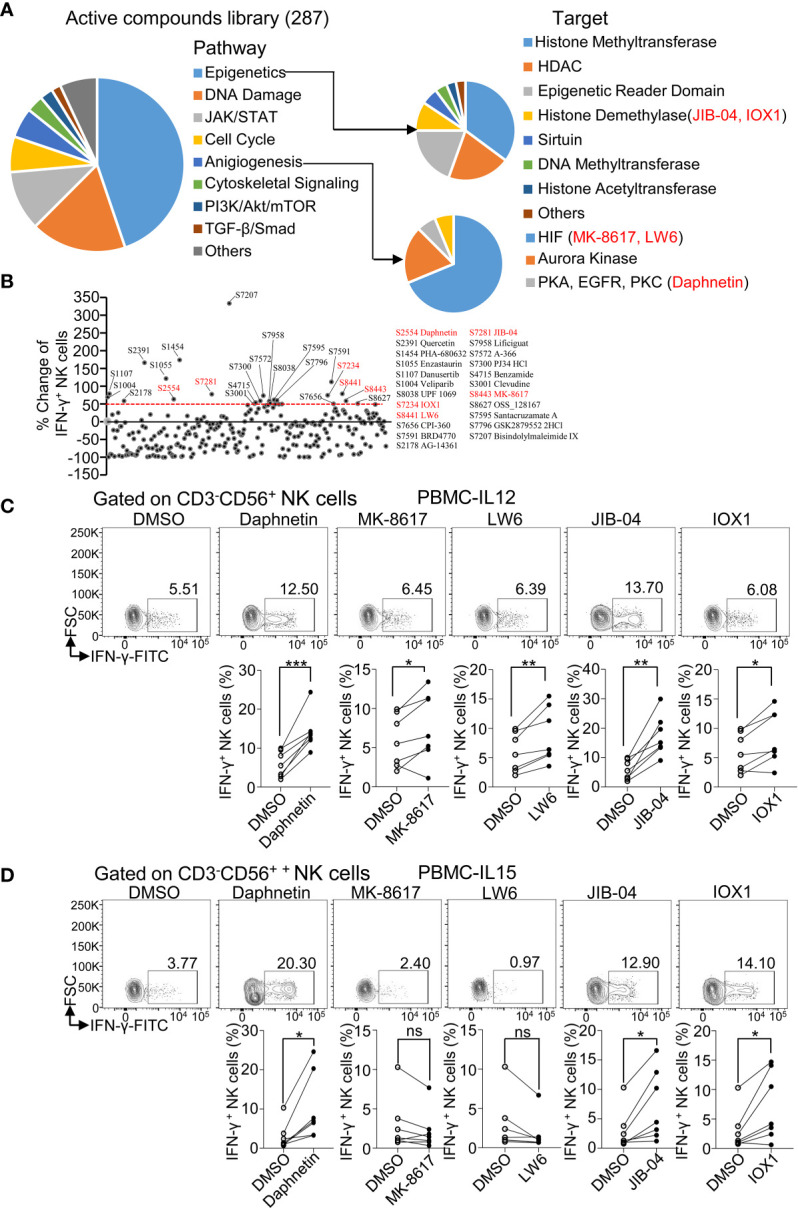
Active compound screening of human NK cell activator *via* determining IFN-γ production. **(A)** Summarization of 287 active compounds based on their molecule targets. **(B)** Distribution of percent change of IFN-γ-producing NK cells treated by the 287 active compounds in the presence of IL-12 (10 ng/ml) for 18 h Twenty-three active compounds that increased more than 50% in IFN-γ^+^ ratio in NK cells than DMSO control were labeled with their catalog numbers. **(C, D)** Flow cytometric analysis and cumulative frequencies of IFN-γ-producing NK cells after healthy donor PBMCs treated by DMSO or indicated active compounds in the presence of IL-12 (10 ng/ml) **(C)** or IL-15 (10 ng/ml) **(D)**, for 18 h Each dot represents one donor. Paired *t*-test was applied between two different treatment and the significant level was shown as *p < 0.05, **p < 0.01, ***p < 0.001, ns, not statistically significant.

A total of 287 candidate compounds from Selleck active compound library were screened for searching purified human primary NK cell activators through determining IFN-γ production. These compounds are involved in the pathways including epigenetics, DNA damage, JAK/STAT, cell cycle, PI3K/Akt/mTOR, and TGF-β/Smad. The targets of these compounds include DNMT, DNA methyltransferase, HDAC, histone demethylase, histone acetyltransferase, PKA, EGFR, and PKC (**Figure 1A**).

IL-12, which is a proinflammatory cytokine produced by various cells and elevated after virus infection or tumorigenesis, is a potent stimulator of NK cell activation and promotes IFN-γ production by NK cells (14). Thus, IL-12 was regarded as a critical element in evaluating the ability of active compounds on NK activation. PBMCs from healthy donors were treated with indicated compounds in the presence of IL-12 (10 ng/ml) for 18 h and then intracellular staining for IFN-γ protein in NK cells was assessed *via* flow cytometric analysis (**Supplementary Figure 1A**). An initial test of one donor per compound revealed that 23 candidates among the 287 active compounds had increased more than 50% in IFN-γ^+^ ratio in NK cells (CD3^-^CD56^+^) compared to the control treatment with IL-12 alone (**Figure 1B** and **Supplementary Table 2**). We next validated these 23 active compounds by using PBMCs from more donors. Daphnetin, MK-8617, LW6, JIB-04, and IOX1 were further selected with the consistent capacity of increasing IFN-γ^+^ ratio in NK cells in almost all tested donors (**Figure 1C**).

IL-15 is critical for NK cell survival and activation, including IFN-γ production (26, 27). Thus, we treated PBMCs from healthy donors with the above five acquired candidates in the presence of IL-15 (10 ng/ml) for 18 h and then measured IFN-γ production. We found that Daphnetin, JIB-04, and IOX1 also increased the IFN-γ^+^ NK cell ratio in the presence of IL-15, whereas LW6 and MK-8617 did not affect IFN-γ production (**Figure 1D**).

We also found that Daphnetin, LW6, and JIB-04 slightly increased the IFN-γ^+^ CD3^+^ T cells ratio in the presence of IL-12 but not in the presence of IL-15 (**Supplementary Figures 1B, C**).

### Effects of the Five Identified Candidates on IFN-γ Secretion in the Supernatants of Cultured PBMCs

**Figure 2:**
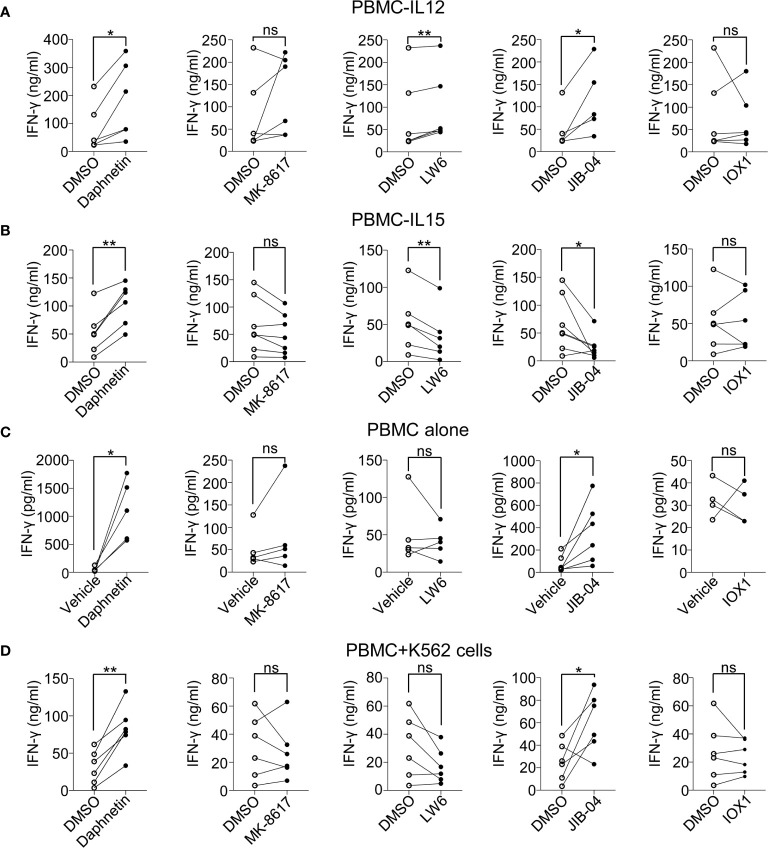
Effects of the five identified candidates on IFN-γ secretion in the supernatants of cultured PBMCs. **(A–C)** Healthy donor PBMCs were treated with DMSO or the identified five candidates for 18 h in the presence of IL-12 (10 ng/ml) **(A)**, IL-15 (10 ng/ml) **(B)**, or in the absence of IL-12 or IL-15 **(C)**, respectively. The protein levels of IFN-γ in the supernatants of cell culture were detected by ELISA kit. **(D)** Healthy donor PBMCs were pre-treated with DMSO or indicated candidates in the presence of IL-12 (10 ng/ml) for 18 h, and co-cultured with K562 cells for another 6 h IFN-γ secretion in the supernatants was detected by ELISA kit. Each dot represents one donor. Paired *t*-test for **(A–D)**. *p < 0.05, **p < 0.01, and ns (not statistically significant) denote statistical comparison between the two marked treatment groups **(A–D)**.

To further determine whether these above five candidates could promote IFN-γ secretion, we next determined the IFN-γ secretion by ELISA in the supernatants of cultured PBMCs 18 h after stimulation in the presence of IL-12 with or without these five candidates. We found that Daphnetin, LW6, and JIB-04 could promote IFN-γ secretion in the presence of IL-12, while MK-8617 and IOX1 did not (**Figure 2A**). Besides, Daphnetin also promoted IFN-γ secretion in the presence of IL-15, whereas MK-8617, LW6, JIB-04, and IOX1 did not or even decreased IFN-γ secretion (**Figure 2B**). We also found that Daphnetin and JIB-04 could promote IFN-γ secretion without IL-12 or IL-15 (**Figure 2C**).

In the presence of IL-12, we also tested the IFN-γ secretion in the cell culture supernatant of PBMCs after co-culturing with K562 cells, which are used as classic target cells of NK cells (28). The data revealed that only Daphnetin and JIB-04 promoted IFN-γ secretion when PBMCs were incubated with K562 cells (**Figure 2D**).

All these lines of evidence suggest different mechanisms of these five candidates in promoting IFN-γ production by human primary NK cells.

### Effects of the Five Identified Candidates on IFN-γ Secretion by Purified Human Primary NK Cells

**Figure 3:**
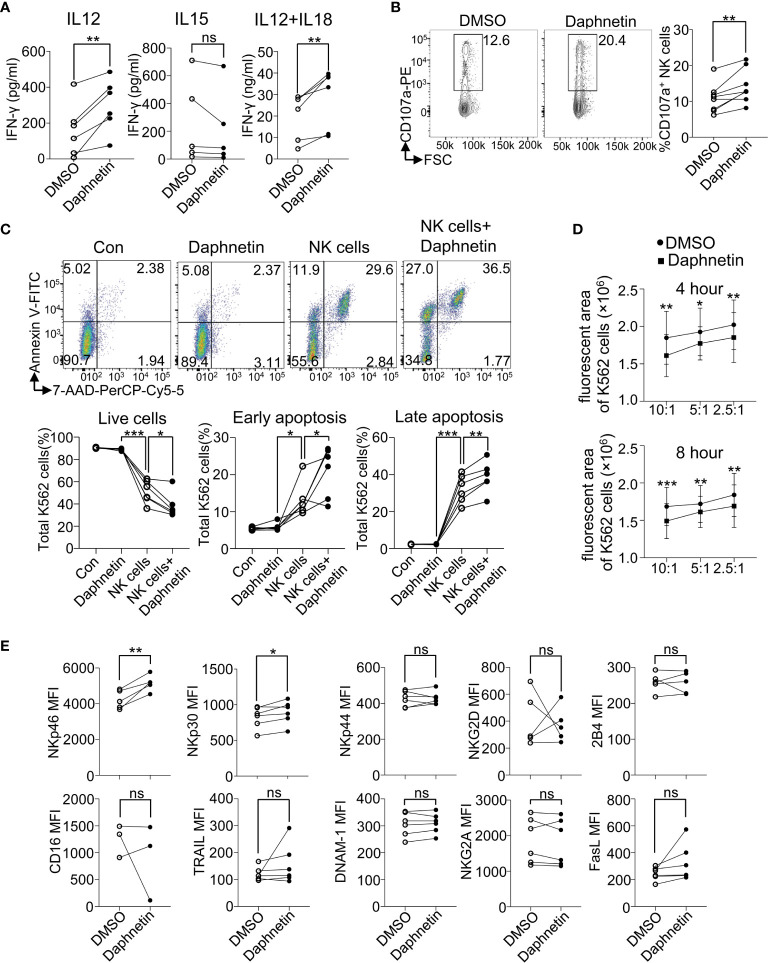
Effects of Daphnetin on IFN-γ secretion and cytotoxicity by purified human primary NK cells. **(A)** Purified human primary NK cells (≥99.0%) were treated with DMSO or Daphnetin for 18 h in the presence of IL-12 (10 ng/ml) ( left), IL-15 (10 ng/ml) (middle ), or IL-12 (10 ng/ml) plus IL-18 (10 ng/ml) (right ), respectively. The levels of IFN-γ in the supernatants of cell culture were detected by ELISA kit. **(B)** Purified human primary NK cells were treated with DMSO or Daphnetin for 18 h in the presence of IL-12 (10 ng/ml), followed by co-culture with K562 cells for another 6 h and the expression level of CD107a on NK cells were determined by flow cytometry. **(C)** The ratio of Annexin V^-^7-AAD^-^, Annexin V^+^7-AAD^-^, and Annexin V^+^7-AAD^+^ K562 cells was determined by flow cytometry after CTV-labeled K562 cells were co-cultured with purified human primary NK cells that were pre-treated with DMSO or Daphnetin in the presence of IL-12 (10 ng/ml). Annexin V^-^ 7-AAD^-^ represents live cells; Annexin V^+^ 7-AAD^-^ represents early apoptosis; Annexin V^+^ 7-AAD^+^ represents late apoptosis. **(D)** The CellTracker dye-labeled K562 cells were imaged by living cell microscopic imaging system after CellTracker dye-labeled K562 cells were co-cultured with purified human primary NK cells treated that pretreated with DMSO or Daphnetin in the presence of IL-12 (10 ng/ml) at the indicated time point. The fluorescent area of K562 cells was recorded and further analyzed using Gen5™ software. **(E)** Purified human primary NK cells (≥99.0%) were treated with DMSO or Daphnetin in the presence of IL-12 (10 ng/ml) for 18 h and the expression of NK activating and inhibitory receptors were detected by flow cytometry. Each dot represents one donor. Paired *t*-test for **(B–E)**. *p < 0.05, **p < 0.01, ***p < 0.001, and ns (not statistically significant) denote statistical comparison between the two marked treatment groups **(B–E)**.

To further distinguish whether these five active compounds enhanced NK cell IFN-γ production directly or indirectly, purified human primary NK cells purified from PBMCs (Purity ≥ 99.0%, **Supplementary Figure 2A**) were co-cultured with the above five candidates in the presence of IL-12, IL-15, and IL-12 + IL-18, respectively. The results showed that Daphnetin enhanced IFN-γ secretion by NK cells in the presence of IL-12 (**Figure 3A**), whereas the other four compounds did not (**Supplementary Figure 2B**). Meanwhile, all these five candidates did not promote NK cell IFN-γ production in the presence of IL-15 (**Figure 3A** and **Supplementary Figure 2C**). In the presence of IL-12 + IL-18, Daphnetin also enhanced NK cell IFN-γ secretion, whereas MK-8617, LW6, JIB-04, and IOX1 had little or even decreased effects on IFN-γ production (**Figure 3A** and **Supplementary Figure 2D**). In addition, Daphnetin did not affect the apoptosis and proliferation of purified human primary NK cells *in vitro* (**Supplementary Figures 3A, B**). These results suggest that Daphnetin promotes NK cell-derived IFN-γ production in a direct manner, which is dependent on IL-12 but not IL-15 signal pathway, whereas MK-8617, LW6, JIB-04, and IOX1 indirectly enhance IFN-γ production in NK cells, which might act on other immune cell subsets, including monocytes and dendritic cells, among PBMCs.

### Effects of Daphnetin on the Cytotoxicity of Human Primary NK Cells

Our above data that Daphnetin enhances NK cell IFN-γ production in the presence of IL-12 in a direct manner prompted us to further explore the role of Daphnetin on NK cell cytotoxicity. CD107a (also called LAMP1) exists in the cytotoxic granular vesicles of NK cells and is transferred to the cell surface during exocytosis when meeting target cells. Thus, the surface expression level of CD107a could reflect the capacity of NK-mediated direct target cell lysis (29). The role of Daphnetin on NK cell cytotoxicity was further explored by the flow cytometric experiment. It revealed that Daphnetin increased the expression of CD107a on purified human primary NK cells when co-cultured with K562 cells in the presence of IL-12 (**Figure 3B**).

To find more evidence that Daphnetin enhances NK cell cytotoxicity, we further verified whether Daphnetin promoted the apoptosis of K562 cells when co-cultured with purified human primary NK cells. Our data showed that Daphnetin itself did not induce K562 cell apoptosis directly; however, it could enhance NK cell-mediated apoptosis of K562 cells in the presence of IL-12, which were evidenced by a decreased ratio of live cells (Annexin V^-^7-AAD^-^), as well as an increased ratio of early apoptosis (Annexin V^+^7-AAD^-^) and late apoptosis (Annexin V^+^7-AAD^+^) of K562 cells after co-culture (**Figure 3C**). Moreover, the enhanced cytotoxicity of NK cells by Daphnetin was also directly observed by living cell microscopic imaging system (**Figure 3D** and **Supplementary Figure 4**).

To further explore whether there is a synergistic effect of IL-12 and Daphnetin on NK cell activation, we next measured IFN-γ secretion and CD107a expression by NK cells from the same donor in different groups with DMSO, Daphnetin, IL-12, and Daphnetin plus IL-12, respectively. The data showed that Daphnetin alone did not affect IFN-γ secretion (**Supplementary Figure 5A**, left panel) and only slightly increased CD107a expression on NK cells without statistical significance (**Supplementary Figure 5B**, left panel). Statistical analysis indicated no synergistic effect of IL-12 and Daphnetin on CD107a expression or IFN-γ secretion (Supplementary **Figures 5A, B**, right panel). Besides, we also determined the expression of activating and inhibitory receptors, including NKp46, NKp30, NKp44, NKG2D, 2B4, CD16, TRAIL, DNAM-1, NKG2A, and FasL, on NK cells treated with Daphnetin in the presence of IL-12. We found that Daphnetin increased the expression of activating receptors (NKp46 and NKp30) (**Figure 3E**).

These findings demonstrate that Daphnetin can enhance the IFN-γ production and direct cytotoxicity of NK cells against tumor cells, which depends on IL-12.

### Mechanisms of Daphnetin in Regulating NK Cell Activation Revealed by RNA-Sequencing Analyses

**Figure 4:**
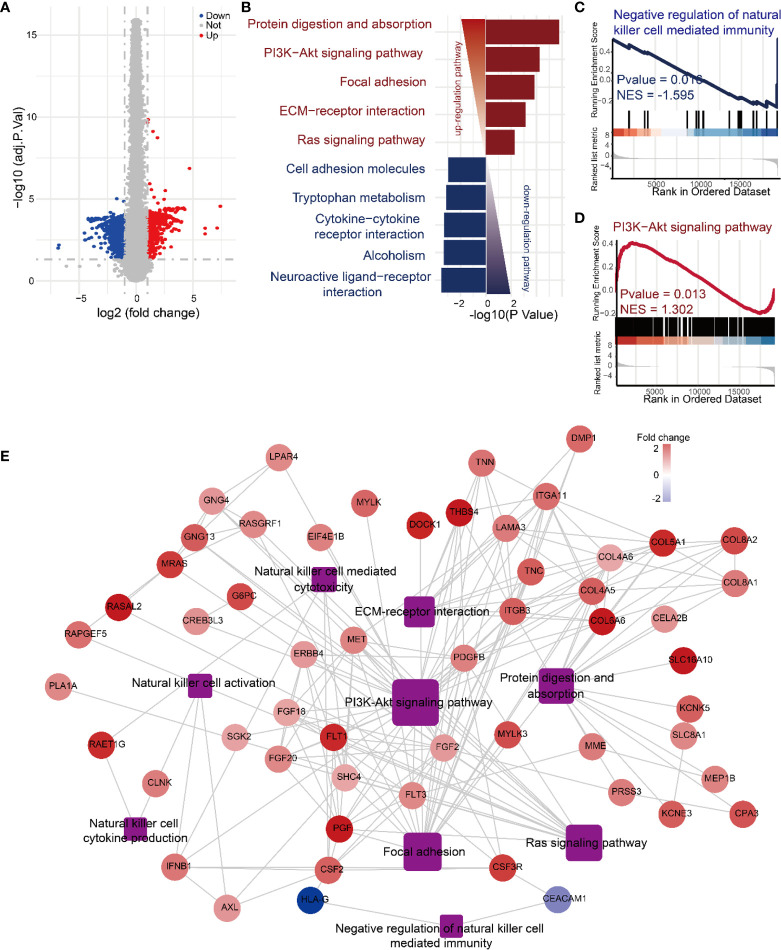
Mechanisms of Daphnetin in regulating NK cell activation by RNA-sequencing analyses. Purified human primary NK cells were treated with DMSO or Daphnetin in the presence of IL-12 (10 ng/ml) for 12 h, followed by RNA-sequencing. **(A)** The volcano plot displayed differentially expressed genes in NK cells between IL-12 and IL-12 plus Daphnetin groups. **(B)** Top five up- and downregulated KEGG pathways of the significant differently expressed genes (DEGs) on NK cells between IL-12 and IL-12 plus Daphnetin groups. Up- and downregulated KEGG pathways are shown in red and blue, respectively. **(C, D)** GSEA analysis of the negative regulation of natural killer cell-mediated immunity signaling pathway **(C)** and PI3K-Akt signaling pathway **(D)** between IL-12 and IL-12 plus Daphnetin groups. **(E)** PPI and gene-pathway network analyses of the genes from the top five upregulated KEGG pathways and NK cell effector function-related DEGs. The ellipse and rectangle show the genes and the gene-related pathways, respectively. The color intensity in each node represents the fold change of the gene in Daphnetin to DMSO samples (upregulation of a gene is shown in red and downregulation of a gene is shown in blue). The size of the rectangle is proportional to the number of genes involved in the pathway.

To further explore the molecular mechanism by which Daphnetin enhances NK cell activation, the differential gene expression between IL-12-treated and IL-12 plus Daphnetin-treated NK cells was further determined by RNA-sequencing. The volcano plot displayed that 786 genes were upregulated and 836 genes were downregulated after Daphnetin treatment (fold change > 1.5) (**Figure 4A**). The top five upregulated KEGG pathways are protein digestion and absorption, PI3K-Akt signaling pathway, focal adhesion, ECM–receptor interaction, and Ras signaling pathway, whereas the top five downregulated KEGG pathways are neuroactive ligand–receptor interaction, alcoholism, cytokine–cytokine receptor interaction, tryptophan metabolism, and cell adhesion molecules (**Figure 4B**). Further GSEA analysis revealed that the negative regulation of NK cell-mediated immunity signals was downregulated and the PI3K-Akt signaling pathway was upregulated in the Daphnetin-treated group, which are similar to the KEGG pathway enrichment results (**Figures 4C, D** and **Supplementary Figure 6**). Moreover, PPI network analysis indicated that the PI3K-Akt signaling pathway had close interactions with other upregulated pathways such as focal adhesion, Ras signaling pathway, protein digestion absorption, ECM–receptor interaction, and NK cell effector function-related signals (**Figure 4E**). These data indicate that PI3K-Akt signaling pathway plays a critical and central role in NK cell activation by Daphnetin.

### PI3K-Akt-mTOR Pathway Participates in the Signal Network of Daphnetin Activated NK Cells

**Figure 5:**
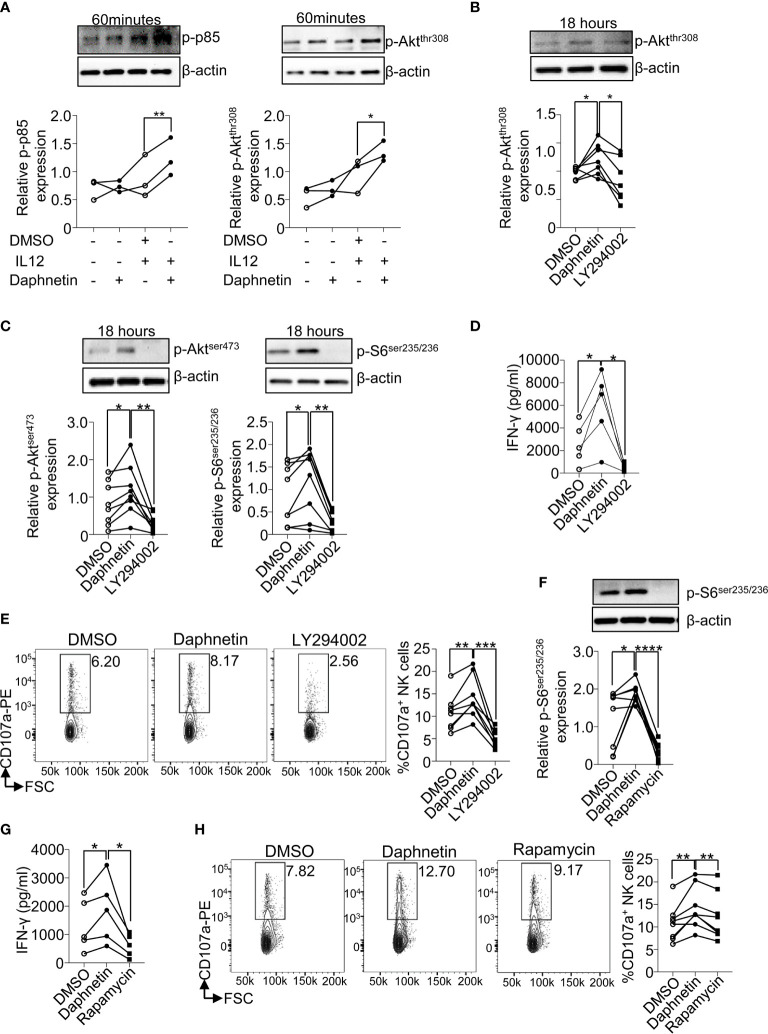
PI3K-mTOR pathway participates in the signal network of Daphnetin-activated NK cells. **(A)** Purified human primary NK cells were treated with DMSO, Daphnetin (10 μM), IL-12 (10 ng/ml), or Daphnetin plus IL-12 for 60 min. The protein levels of p-p85 (left ) and p-Akt^thr308^ (right ) in NK cells were determined by immunoblotting. **(B–D)** Purified human primary NK cells were treated with DMSO or LY294002 (10 μM) in the presence of IL-12 (10 ng/ml) and Daphnetin (10 μM) for 18 h The protein levels of p-Akt ^thr308^ **(B)**, p-Akt^ser473^ (**C**, left ), and p-S6^ser235/236^ (**C**, right ) in NK cells were determined by immunoblotting, and IFN-γ secretion in the supernatants of cell culture **(D)** was determined by ELISA, respectively. **(E)** Purified human primary NK cells were treated with DMSO or LY294002 (10 μM) in the presence of IL-12 (10 ng/ml) and Daphnetin (10 μM) for 18 h, and then co-cultured with K562 cells for another 6 h, and the expression level of CD107a on NK cells was determined by FACS. **(F, G)** Purified human primary NK cells were treated with DMSO or rapamycin (1 nM) in the presence of IL-12 (10 ng/ml) and Daphnetin (10 μM) for 18 h The protein level of p-S6^ser235/236^ **(F)** and IFN-γ secretion in the supernatants of cell culture **(G)** were determined by immunoblotting and ELISA, respectively. **(H)** Purified human primary NK cells were treated with DMSO or rapamycin (1 nM) in the presence of IL-12 (10 ng/ml) and Daphnetin (10 μM) for 18 h, and then co-cultured with K562 cells for another 6 h and the expression level of CD107a on NK cells was determined by FACS. Paired *t*-test for **(A–H)**. *p < 0.05, **p < 0.01, ***p < 0.001,****p < 0.0001 **(A–H)**.

Previous studies have demonstrated that PI3K-Akt-mTOR plays a pivotal role in the regulation of NK cell activation and effector functions (26, 27). When PI3K signaling pathway is activated by various factors, its regulatory subunit p85 is phosphorylated and promotes the phosphorylation of Akt at thr308 (p-Akt^thr308^), which results in the activation of mTOR complex (mTORC) 1 and mTORC2, indicated by the phosphorylation of S6 at ser235/236 (p-S6^ser235/236^) and Akt at ser473 (p-Akt^ser473^), respectively (30). Inhibition of PI3K signaling pathway in NK cells interferes with perforin and granzyme B movement toward target cells and suppresses NK cell cytotoxicity (31). Deletion of PI3K class IB and class IA catalytic subunits (p110γ and p110δ) or mTOR prevents full NK cell maturation, IFN-γ production, and cytotoxicity (32). Our data showed that Daphnetin alone showed comparable levels of p-p85 and p-Akt^thr308^ with the control group; however, Daphnetin increased the levels of p-p85 and p-Akt^thr308^ in the presence of IL-12, compared with IL-12 alone after 60 min of treatment (**Figure 5A**). Our data also showed that IL-12 only showed a slightly increased levels of p-p85 and p-Akt^thr308^ by NK cells without statistical significance with control, which was consistent with the previous finding in murine NK cells (27). To further confirm the role of the PI3K-Akt signaling pathway in NK cell activation by Daphnetin, the PI3K-Akt signaling pathway inhibitor LY294002 was applied. Our data showed that compared with IL-12 alone, Daphnetin showed increased levels of p-Akt^thr308^ in NK cells in the presence of IL-12, which was reversed by LY294002 after 18 h treatment (**Figure 5B**). As referred to mTORC1 and mTORC2 signaling, both the levels of p-S6^ser235/236^ and p-Akt^ser473^ were enhanced by Daphnetin in purified human primary NK cells, which were reversed by LY294002 in the presence of IL-12 (**Figure 5C**). As expected, increased IFN-γ secretion and CD107a expression on NK cells by Daphnetin were reversed by LY294002 in the presence of IL-12 (**Figures 5D, E**). We also confirmed these findings by using PBMCs co-cultured with Daphnetin in the presence or absence of LY294002 (**Supplementary Figures 7A, B**).

mTORC1 is the main downstream of the PI3K-Akt signaling pathway (33), and our previous study also found that NK cells with mTORC1-specific deficiency had impaired IFN-γ production (17). To investigate whether Daphnetin enhanced IFN-γ production in NK cells *via* the mTORC1 signaling pathway, the mTORC1 inhibitor rapamycin was applied. The data showed that rapamycin treatment could reverse the levels of p-S6^ser235/236^ and also the IFN-γ production and CD107a expression by Daphnetin, irrespective of using purified human primary NK cells or PMBC co-culture (**Figures 5F–H** and **Supplementary Figures 7C, D**).

In order to explore whether Daphnetin enhances signaling events downstream of IL-12, we also compared the phosphorylation levels of STATs between DMSO and Daphnetin in the presence of IL-12. The data showed that the levels of p-STAT3, p-STAT4, and p-STAT5 were not significantly changed after Daphnetin treatment (**Supplementary Figure 8**).

Together, we demonstrate that the PI3K-Akt-mTOR signaling pathway is involved in the regulation of Daphnetin-mediated IFN-γ production in purified human primary NK cells

## Discussion

NK cells have a strong and quick capacity to kill tumor cells and clear viral infection; thus, enhancement of NK cell activity is an attractive approach to the treatment of cancer and viral infection (10). In this study, through active compound screening, we identified Daphnetin (34), a dihydroxylated derivative of coumarin, as a potent stimulator of human NK cells by enhancing IFN-γ production and direct cytotoxicity in the presence of IL-12. Previous studies have revealed that Daphnetin exerts anti-inflammatory and anti-oxidant activities, and also directs cancer cell death. For example, in the context of chronic inflammatory status, Daphnetin could inhibit the activation of T helper (Th) 1, 2, and 17 cells, but increase the ratio of regulatory T (Treg) cells. Daphnetin could regulate the balance of Th17 and Treg cells in the PMBCs from patients with unexplained recurrent pregnancy loss (35), in the intestine of dextran sodium sulfate-induced experimental colitis in mice (36) and also in collagen-induced arthritis in rats (37, 38). Daphnetin also suppresses the production of inflammatory cytokines in experimental autoimmune encephalomyelitis mice (39). In the context of cancer, Daphnetin could induce the direct death of cancer cells (34, 40, 41). Here, our study opens up a new potential application for Daphnetin as a complementary immunomodulator for cancer treatments.

For inducing NK cell activation, IL-12 mainly utilizes the JAK/STAT pathway, whereas IL-15 mainly uses the PI3K-Akt-mTOR pathway (26, 27, 42). We found that Daphnetin enhanced the role of IL-12, but not IL-15, in promoting IFN-γ production and cytotoxicity by purified human primary NK cells. Interestingly, our data revealed that Daphnetin enhanced PI3K-Akt-mTOR but not STAT signaling in the presence of IL-12. This seems not to be consistent with the finding that Daphnetin did not increase NK cell IFN-γ production in the presence of IL-15, which mainly uses PI3K-Akt-mTOR signaling. One potential explanation is that the capacity of Daphnetin on activating PI3K-Akt-mTOR signaling is not as strong as IL-15 in NK cells. IL-15 could dramatically activate the PI3K-Akt-mTOR signaling pathway, 2.5- to 4-fold higher than untreated NK cells. When used combined with IL-15, its effect was covered up by IL-15. Although IL-12 alone only showed slightly increased levels of p-p85 and p-Akt^thr308^, IL-12 plus Daphnetin resulted in significantly enhanced levels of p-p85 and p-Akt^thr308^ by NK cells. This suggests that IL-12 alone could not fully activate the PI3K-Akt signaling pathway in the aspect of IFN-γ secretion and cytotoxicity, which agreed with the previous study that IL-12 alone did not dramatically activate mTOR pathway as that by IL-15 in NK cells (27). However, the combination of IL-12 and Daphnetin breaks through the threshold of PI3K-Akt signaling pathway activation, resulting in augmented IFN-γ secretion and cytotoxicity by NK cells. Full activation of PI3K-Akt is involved in both human and murine NK cell IFN-γ secretion and cytotoxicity (27, 43). A similar phenomenon also exists in T cells; PI3K-Akt signaling is responsible for IL-12-mediated proliferation and survival signals but not IFN-γ production by CD4^+^ T cells (44). The mechanisms of Daphnetin regulating PI3K-Akt-mTOR signaling in NK cells need further detailed research.

In our study, Daphnetin enhanced PI3K-Akt-mTOR signaling in the presence of IL-12. However, several studies have revealed that Daphnetin represses PI3K-Akt signaling, which exerts anti-inflammatory and direct cancer cell death (34, 40, 41, 45). The dichotomous role of Daphnetin in regulating PI3K-Akt-mTOR signaling is interesting. Firstly, the concentration or dose of Daphnetin used in these studies should be considered. Inhibition of PI3K-Akt signaling by Daphnetin induces direct cancer cell death, which is based on much higher concentration use of Daphnetin (34, 40, 41, 45). At the concentration of Daphnetin (10 μM) we used, it did not induce K562 cells apoptosis alone. Another reason for this discrepant regulation of PI3K-Akt signaling might be the different ATP levels in various cell types or even the same cell type with different statuses. Yang et al. have demonstrated that the inhibition of protein kinase activity by Daphnetin is competitive toward ATP. Daphnetin could inhibit EGF-induced tyrosine phosphorylation of EGF receptor in the presence of ATP *in vitro*, whereas it could not repress EGF-induced tyrosine phosphorylation of EGF receptor in human hepatocellular carcinoma HepG2 cells (46). However, this is still a very complex issue that needs further in-depth study.

In summary, we identify a natural compound, Daphnetin, which effectively activates NK cells effector functions by activating the PI3K-Akt-mTOR signaling pathway in the presence of IL-12. This current study adds PI3K-Akt-mTOR as an additional target for Daphnetin. Further studies exploring the possibility of combined therapy of Daphnetin and NK cell-based immunotherapy against cancer, especially optimizing the dose used *in vivo*, are warranted.

## Data Availability Statement

The original contributions presented in the study are publicly available. These data can be found here: The National Genomics Data Center (https://ngdc.cncb.ac.cn/gsa-human/browse/HRA000968).

## Ethics Statement

The studies involving human participants were reviewed and approved by the Institutional Review Board of Hunan Children’s Hospital, authorization number HCHLL-2019-48. The ethics committee waived the requirement of written informed consent for participation.

## Author Contributions

The work presented was performed in collaboration with all authors. BY designed and performed the experiments, analyzed the data, and wrote the manuscript. QY and YY performed the experiments and analyzed the data. YL, HP, SW, LW, SZ, MH, EW, PX, and TL performed the experiments. LL and SJ designed the research and supervised the study. YaD designed the research, analyzed the data, and wrote the manuscript. YoD devised the concept, designed the research, supervised the study, and wrote the manuscript. All authors contributed to the article and approved the submitted version.

## Conflict of Interest

The authors declare that the research was conducted in the absence of any commercial or financial relationships that could be construed as a potential conflict of interest.

## Publisher’s Note

All claims expressed in this article are solely those of the authors and do not necessarily represent those of their affiliated organizations, or those of the publisher, the editors and the reviewers. Any product that may be evaluated in this article, or claim that may be made by its manufacturer, is not guaranteed or endorsed by the publisher.
