# Supplementary material for: Screening for Active Compounds Targeting Human Natural Killer Cell Activation Identifying Daphnetin as an Enhancer for IFN-γ Production and Direct Cytotoxicity
Source: Front Immunol. 2021 Dec 8;12:680611. doi: 10.3389/fimmu.2021.680611 (PMC8693168; doi:10.3389/fimmu.2021.680611)
Supplement: Supplementary file 1 [file DataSheet_1.docx]

**Screening for Active Compounds Targeting Human Natural Killer Cell Activation Identifying Daphnetin as An Enhancer for IFN-γ Production and Direct Cytotoxicity**

Baige Yao^1,2*^, Qinglan Yang^2,3,*^, Yao Yang^4^, Yana Li^2,3^, Hongyan Peng^2,3^, Shuting Wu^2,3^, Lili Wang^2,3^, Shuju Zhang^2,3^, Minghui Huang^2,3^, ErqiangWang^2,3^, Peiwen Xiong^2,3^, Ting Luo^2,3^, Liping Li^2,3,#^, Sujie Jia^1,#^, Yafei Deng^2,3,#^, Youcai Deng^4,#^

^1^Department of Pharmacy, The Third Xiangya Hospital, Central South University, Changsha, 410013, Hunan, China;

^2^Hunan Children’s Research Institute (HCRI), Hunan Children’s Hospital, Changsha, China;

^3^Hunan Provincial Key Laboratory of Children's Emergency Medicine, Hunan Children's Hospital, Changsha, China;

^4^Institute of Materia Medica, College of Pharmacy, Army Medical University (Third Military Medical University), Chongqing 400038, China;

*Co-first author

^#^Correspondence: Youcai Deng (youcai.deng@tmmu.edu.cn); Yafei Deng (yafeideng01@sina.com); Sujie Jia (sujiejia@csu.edu.cn); Liping Li (13974871993@163.com).

**Numbers of words: 8710**

**Numbers of figures: 5**

**Running title:** Daphnetin as a Potent NK Activator

**Supplemental Figure and Figure legends**

**
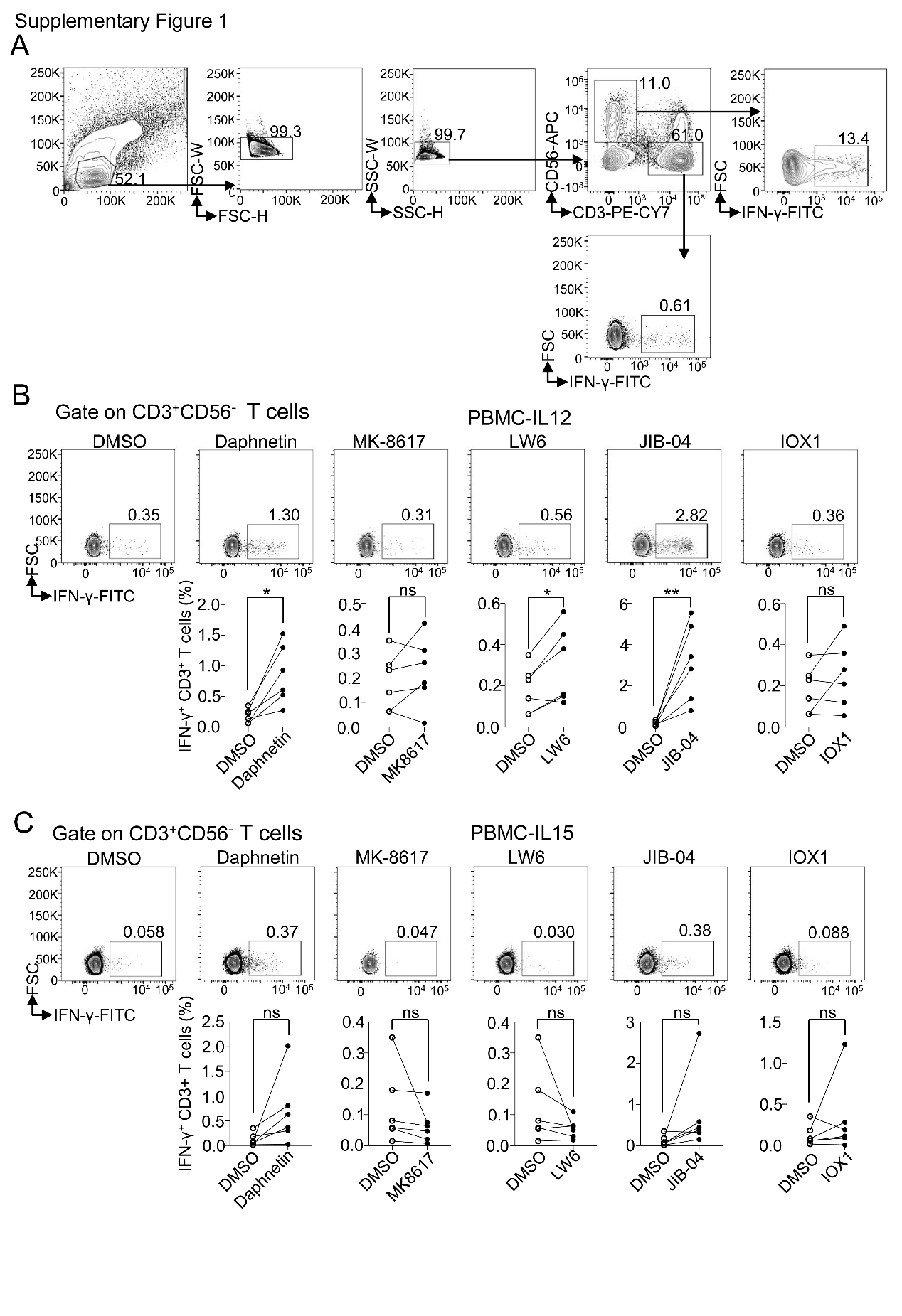
Supplemental Figure 1.** Effects of the five identified candidates on IFN-γ Secretion of CD3^+^ T cells. (**A**) Gating strategy of flow cytometry of IFN-γ ratio in CD3^+^ CD56^-^ T cell. (**B and** **C**) Flow cytometric analysis and cumulative frequencies of IFN-γ-producing CD3^+^ CD56^-^ T cells after healthy donor PBMCs treated by DMSO or the indicated compounds in the presence of IL-12 (10 ng/ml) (**B**) or IL-15 (10 ng/ml) (**C**), for 18 hours. Each dot represents one donor. Paired t-test for **B** and **C**. **p* < 0.05, ***p* < 0.01, and ns (no statistical significant) denotes statistical comparison between the two marked treatment groups (**B** **and** **C**).


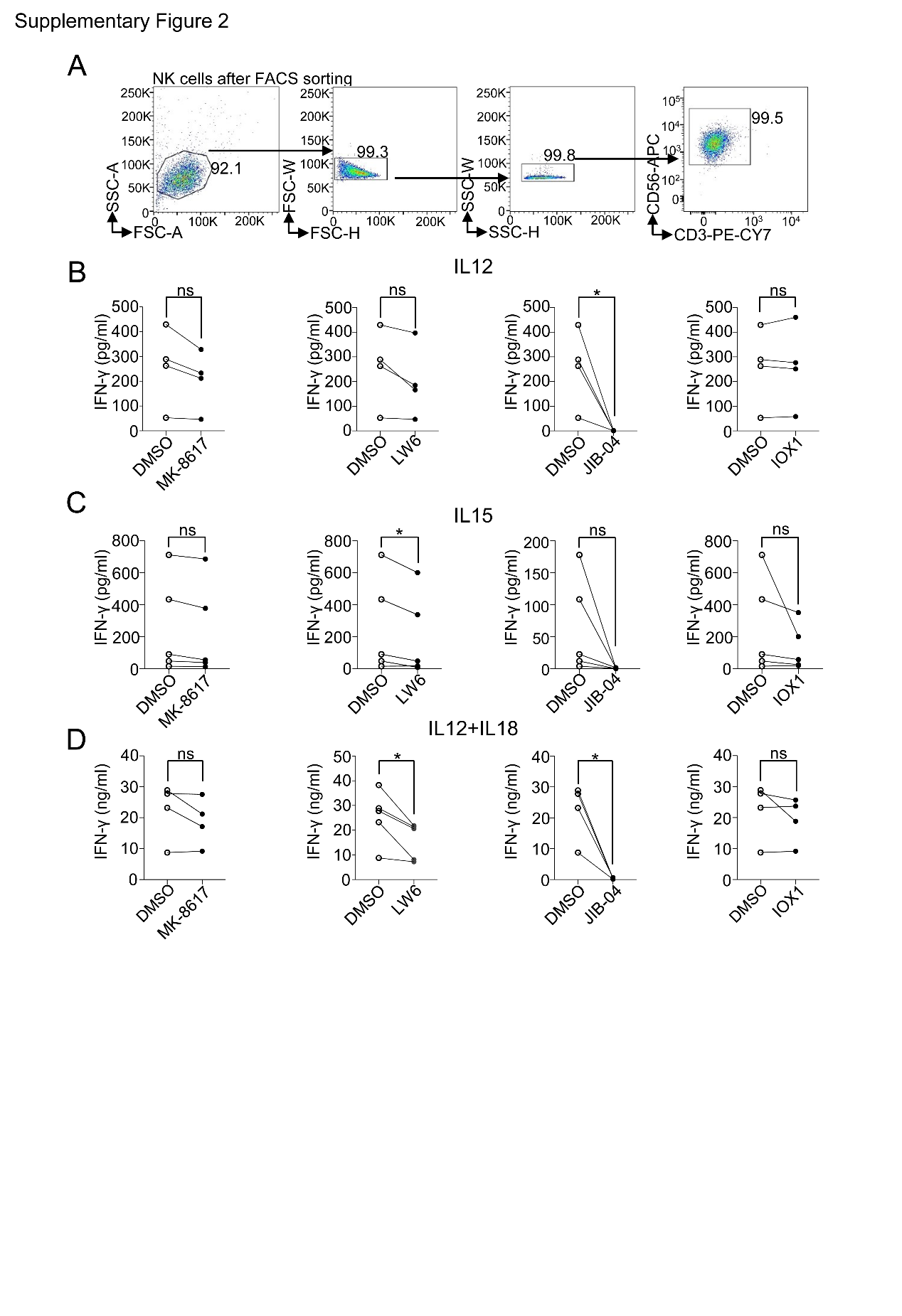


**Supplemental Figure 2.** Effects of the four identified candidates on IFN-γ secretion of purified human primary NK cells. (**A**) Representative figure of purified human primary NK cells purity after FACS sorting. (**B-D**) Purified human primary NK cells were treated with DMSO or indicated candidates for 18 hours in the presence of IL-12 (10 ng/ml) (**B**), IL-15 (10 ng/ml) (**C**), or IL-12 (10 ng/ml) plus IL-18 (10 ng/ml) (**D**), respectively. The levels of IFN-γ in the supernatants of cell culture were detected by ELISA kit.

**
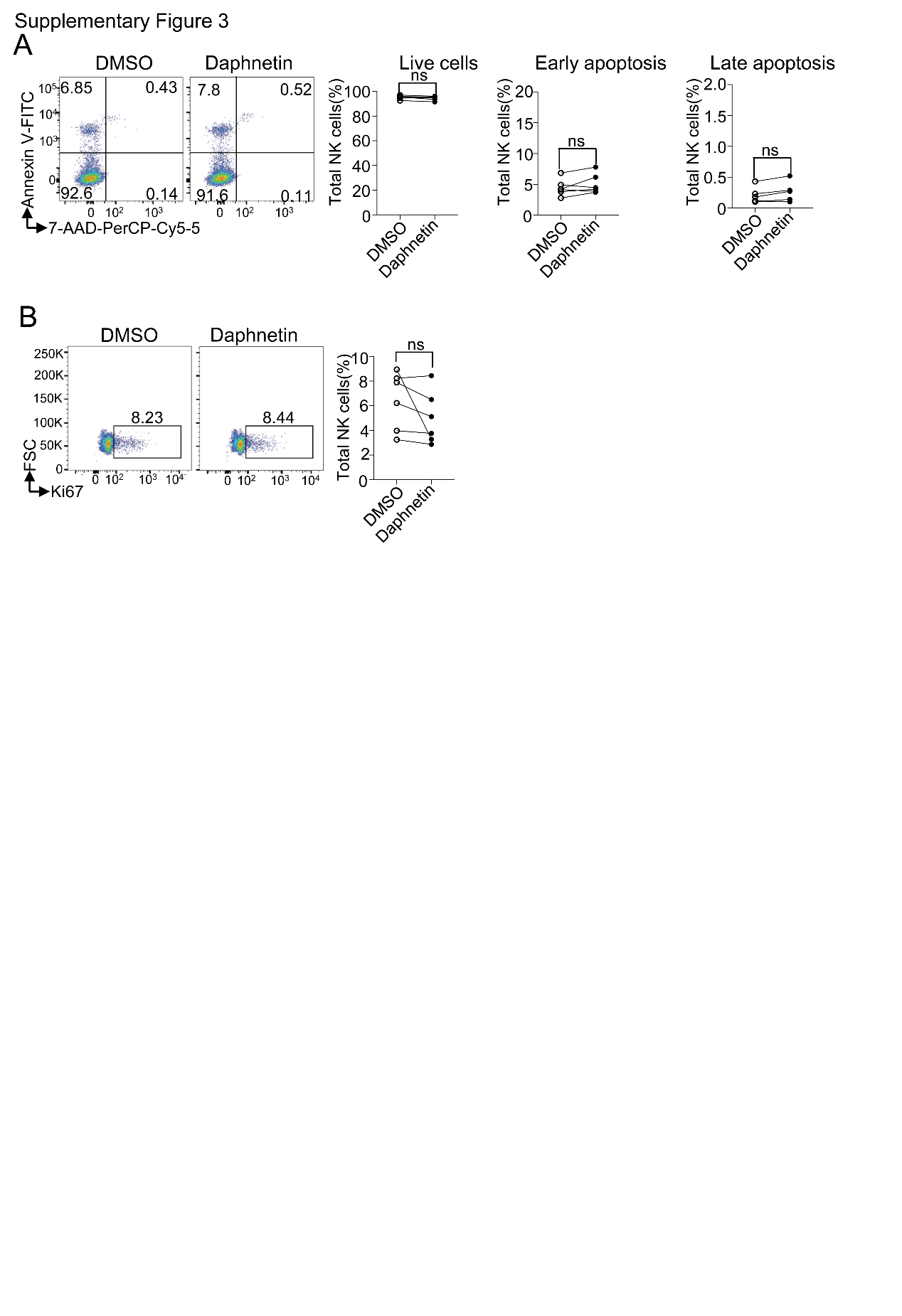
**

**Supplemental Figure 3.** Effects of Daphnetin on the apoptosis and proliferation of purified human primary NK cells. **(A-B**) Purified human primary NK cells were treated with DMSO or Daphnetin for 18 hours in the presence of IL-12 (10 ng/ml). The ratio of Annexin V^-^7-AAD^-^, Annexin V^+^7-AAD^-^, Annexin V^+^7-AAD^+^ NK cells (**A**) and the ratio of Ki67^+^ NK cells were determined by flow cytometry, respectively. Annexin V^-^ 7-AAD^-^ represents live cells; Annexin V^+^ 7-AAD^-^ represents early apoptosis; Annexin V^+^ 7-AAD^+^ represents late apoptosis. ns (no statistical significant) denotes statistical comparison between the two marked treatment groups (**A** **and** **B**).


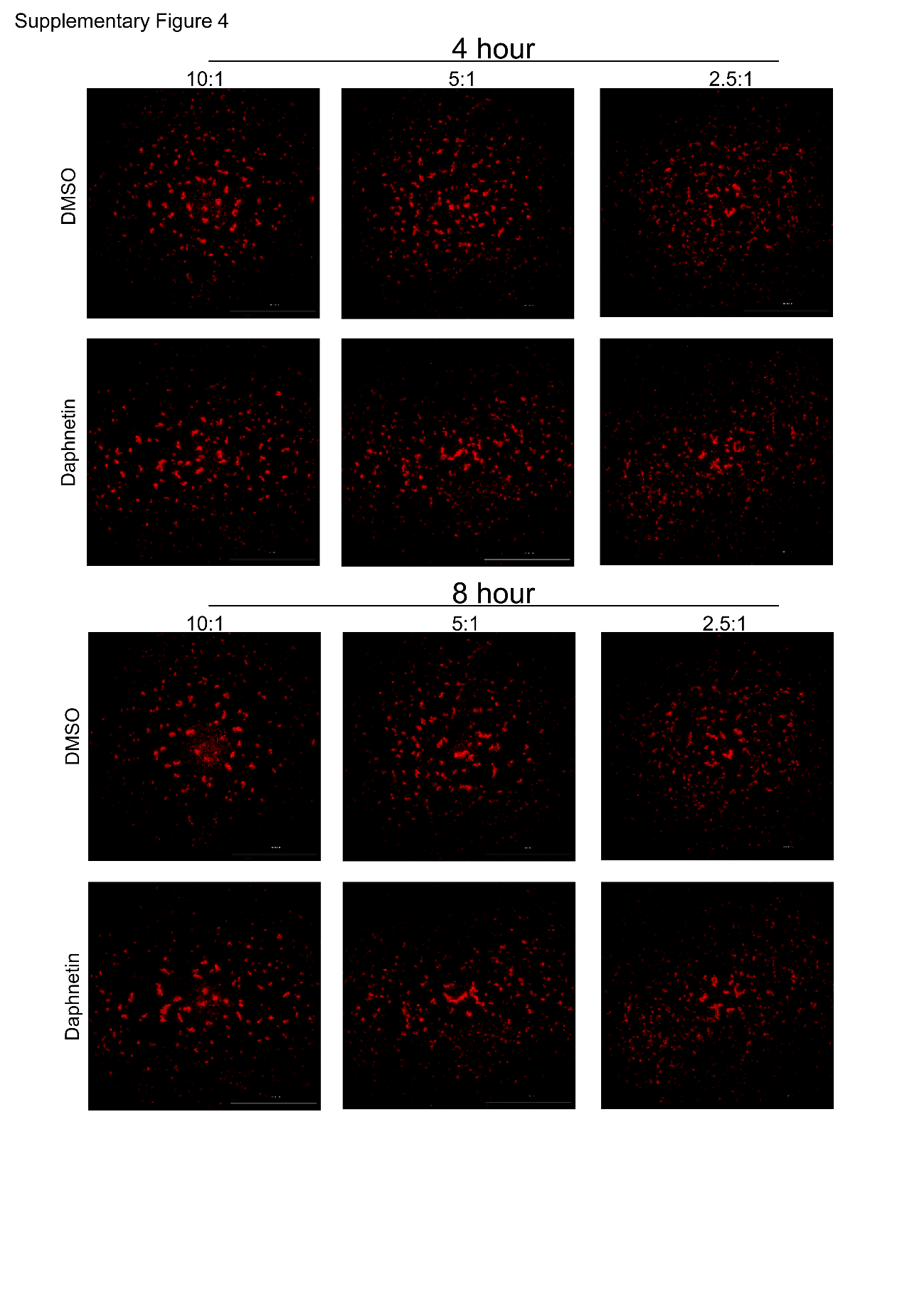


**Supplemental Figure 4.** Representative picture of CellTracker dye-labeled K562 cells after co-cultured with purified human primary NK cells. Representative picture of CellTracker dye-labeled K562 cells after co-cultured with purified human primary NK cells for 4 or 8 hours in Figure 3D. The fluorescent area of K562 cells was recorded and further analyzed using Gen5^TM^ software.


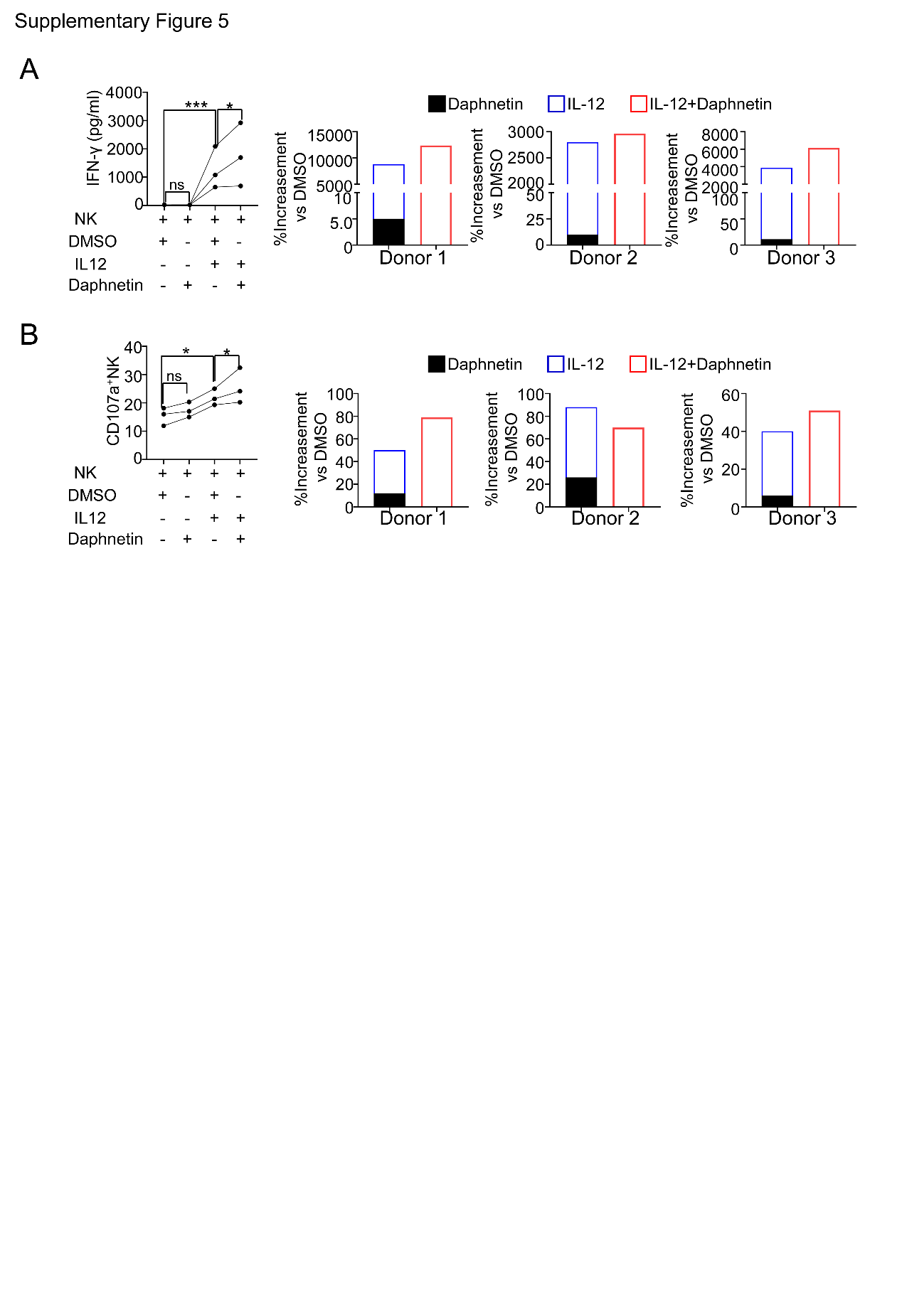


**Supplemental Figure 5.** Synergistic effect of IL-12 and Daphnetin on IFN-γ and CD107a induction by purified human primary NK cells. (**A-B**) NK cells were treated as described in Figure 3A-B, IFN-γ in the supernatant and CD107a were determined by ELISA (**A**) and flow cytometry (**B**), respectively. The increase of IFN-γ in each treatment compared to the control treatment with DMSO is presented in percentage. In each donor, the paired bars compare the additive effect of IL-12 and Daphnetin -treated alone (left, composite bar) versus the effect of the co-stimulation with IL-12 and Daphnetin (right, black bar). Additive effect of IL-12 and Daphnetin versus co-stimulation with IL-12 and Daphnetin. *p* > 0.05.


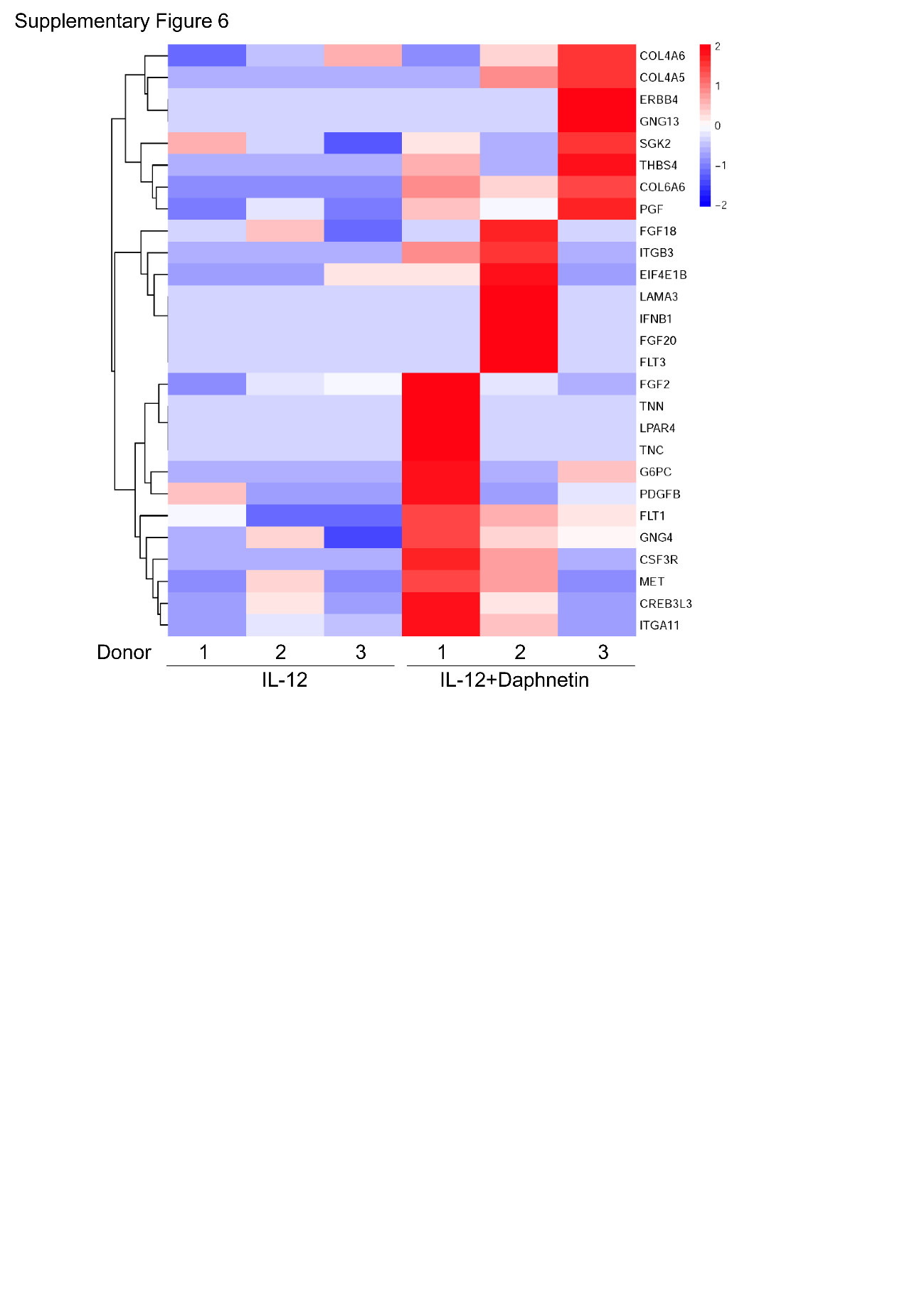


**Supplemental Figure 6.** Heatmap of significantly changed genes in PI3K-Akt pathway. Purified human primary NK cells were treated with DMSO or Daphnetin in the presence of IL-12 (10 ng/ml) for 12 hours, followed by RNA-sequencing (n=3). Heatmap displaying expression of significantly changed genes in PI3-Atk signaling pathway on individual donors treated with IL-12 or IL12 plus Daphnetin. Paired t tests were used to compare samples.


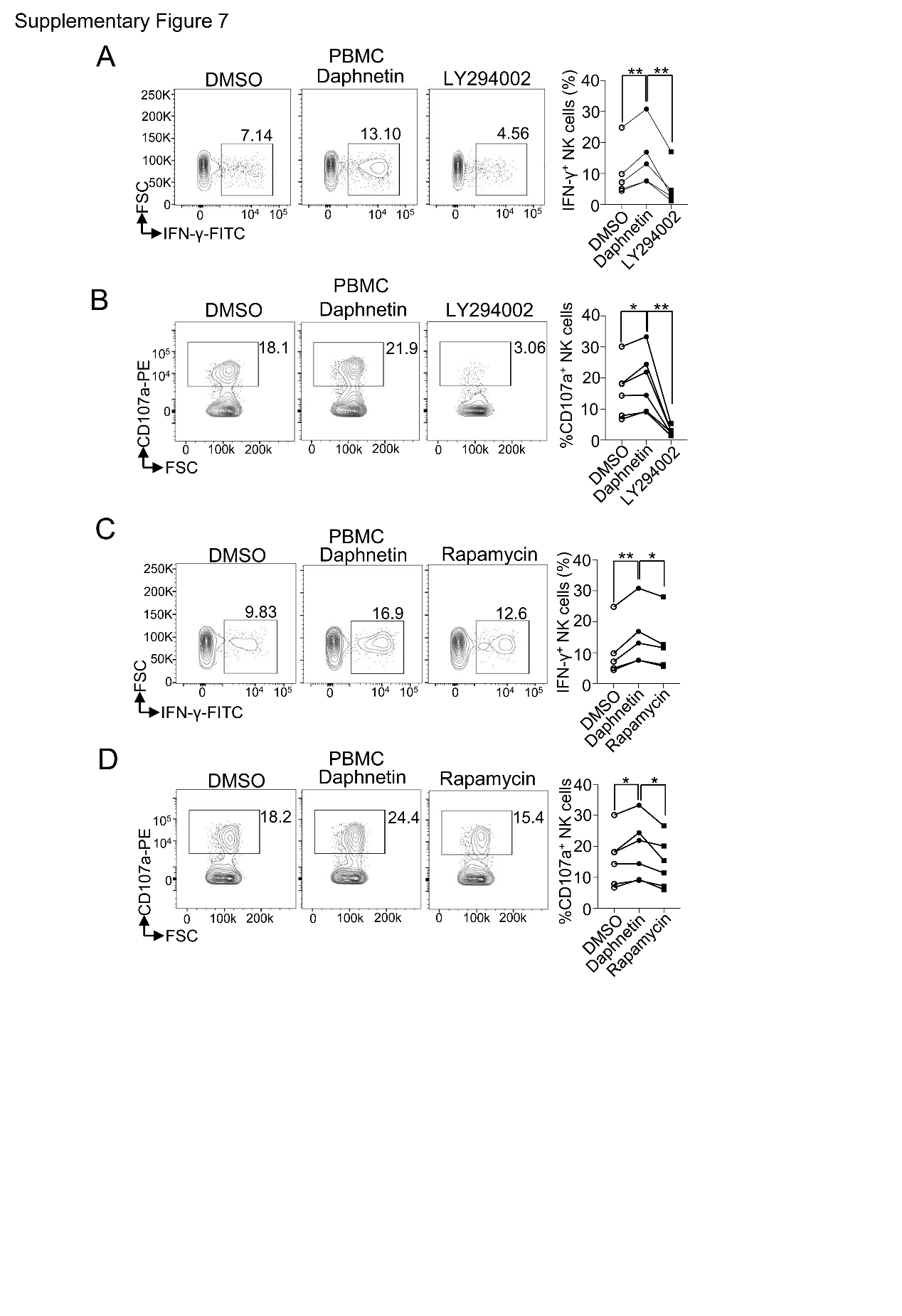


**Supplemental Figure 7.** PI3K-mTOR pathway participates in the signal network of Daphnetin activated NK cells. (**A**) Healthy donor PBMCs were treated with DMSO or LY294002 (10 μM) in the presence of IL-12 (10 ng/ml) and Daphnetin (10 μM) for 18 hours. IFN-γ secretion in the supernatants of cell culture was determined by ELISA. (**B**) Healthy donor PBMCs were treated with DMSO or LY294002 (10 μM) in the presence of IL-12 (10 ng/ml) and Daphnetin (10 μM) for 18 hours, followed by co-cultured with K562 cells for another 6 hours and the expression level of CD107a on NK cells was determined by FACS. (**C**) Healthy donor PBMCs were treated with DMSO or rapamycin (1 nM) in the presence of IL-12 (10 ng/ml) and Daphnetin (10 μM) for 18 hours. IFN-γ secretion in the supernatants of cell culture was determined by ELISA. (**D**) Healthy donor PBMCs were treated with DMSO or rapamycin (1 nM) in the presence of IL-12 (10 ng/ml) and Daphnetin (10 μM) for 18 hours, followed by co-cultured with K562 cells for another 6 hours and the expression level of CD107a on NK cells was determined by FACS. Paired t-test for **A**-**D**. **p* < 0.05, ***p* < 0.01 denotes statistical comparison between the two marked treatment groups (**A-D**).


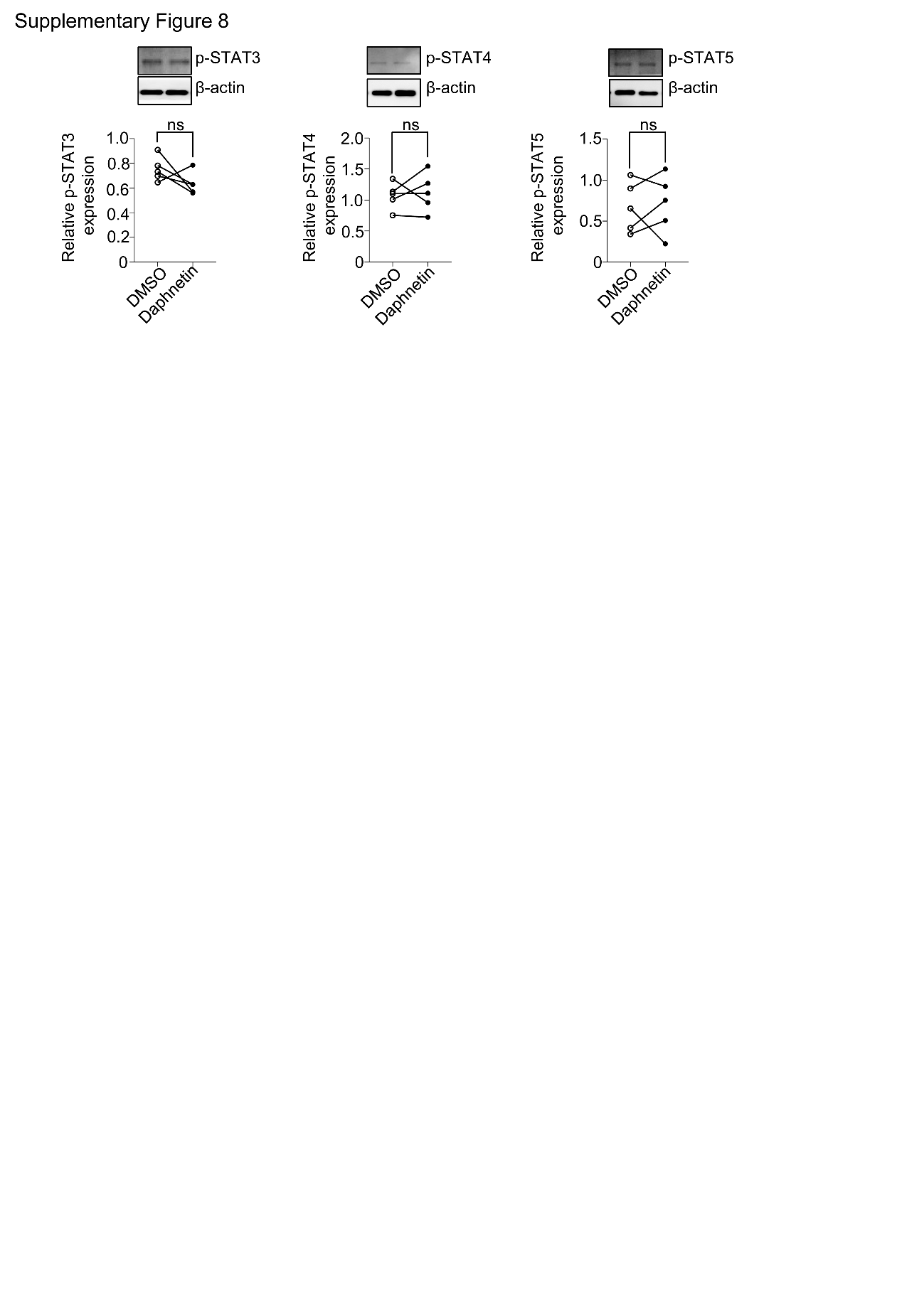


**Supplemental Figure 8.** Effects of Daphnetin on the phosphorylation levels of STATs in purified human primary NK cells. Purified human primary NK cells were treated with DMSO or Daphnetin for 18 hours in the presence of IL-12 (10 ng/ml). The protein levels of p-STAT3 (**left**), p-STAT4 (**middle**) and p-STAT5 (**right**) in NK cells were determined by immunoblotting. ns (no statistical significant) denotes statistical comparison between the two marked treatment groups.
