# Supplementary material for: Screening for Active Compounds Targeting Human Natural Killer Cell Activation Identifying Daphnetin as an Enhancer for IFN-γ Production and Direct Cytotoxicity
Source: Front Immunol. 2021 Dec 8;12:680611. doi: 10.3389/fimmu.2021.680611 (PMC8693168; doi:10.3389/fimmu.2021.680611)
Supplement: Supplementary file 2 [file Table_1.docx]

Supplemental Table 1. Antibodies used for flow cytometry and immunoblotting.

| Antibodies | Clone | Source | Dilution |
| --- | --- | --- | --- |
| anti-human CD3 | HIT3a | BioLegend | 1/400 |
| anti-human CD56 | QA17A16 | BioLegend | 1/400 |
| anti-human IFN-γ | 4S.B3 | BD Biosciences | 1/400 |
| anti-human CD107a | H4A3 | BD Biosciences | 1/120 |
| anti-human NKp46 | 9E2 | BioLegend | 1/400 |
| anti-human NKp30 | REA823 | Miitenyi Biotec | 1/400 |
| anti-human NKp44 | P44-8 | BioLegend | 1/400 |
| anti-human NKG2D | 1D11 | BD Biosciences | 1/400 |
| anti-human NKp46 | 9E2 | BioLegend | 1/400 |
| anti-human 2B4 | 2-69 | BD Biosciences | 1/400 |
| anti-human FasL | REA1056 | Miitenyi Biotec | 1/400 |
| anti-human CD16 | 3G8 | BioLegend | 1/400 |
| anti-human TRAIL | RIK-2 | BD Biosciences | 1/400 |
| anti-human DNAM-1 | DX11 | BD Biosciences | 1/400 |
| anti-human NKG2A | REA110 | Miitenyi Biotec | 1/400 |
| Ki67 | SolA15 | eBioscience | 1/1000 |
| Annexin V | / | BioLegend | 1/20 |
| 7-AAD | / | BioLegend | 1/20 |
| p-p85 | / | Cell Signaling Technology | 1/1000 |
| p-STA3 | D3A7 | Cell Signaling Technology | 1/1000 |
| p-STA4 | D2E4 | Cell Signaling Technology | 1/1000 |
| p-STA5 | C71E5 | Cell Signaling Technology | 1/1000 |
| p-Akt^ser473^ | D9E | Cell Signaling Technology | 1/1000 |
| p-Akt^thr308^ | 244F9 | Cell Signaling Technology | 1/1000 |
| p-S6^ser235/236^ | D57.2.2E | Cell Signaling Technology | 1/1000 |
| β-actin | 8H10D10 | Cell Signaling Technology | 1/5000 |
