# Supplementary material for: Screening for Active Compounds Targeting Human Natural Killer Cell Activation Identifying Daphnetin as an Enhancer for IFN-γ Production and Direct Cytotoxicity
Source: Front Immunol. 2021 Dec 8;12:680611. doi: 10.3389/fimmu.2021.680611 (PMC8693168; doi:10.3389/fimmu.2021.680611)
Supplement: Supplementary file 3 [file Table_2.docx]

Supplemental Table 2. Percentage of IFN-γ-producing NK cells treated by active compounds in the presence of IL-12.

| Catalog Number | Product Name | Percentage of IFN-γ | Catalog Number | Product Name | Percentage of IFN-γ |
| --- | --- | --- | --- | --- | --- |
| S7207 | Bisindolylmaleimide IX (Ro 31-8220 Mesylate) | 333.91 | S5438 | Biphenyl-4-sulfonyl chloride | 31.32 |
| S1454 | PHA-680632 | 174.11 | S7792 | SRT2104 (GSK2245840) | 29.84 |
| S2391 | Quercetin | 166.53 | S5445 | AMI-1, free acid | 29.84 |
| S1055 | Enzastaurin (LY317615) | 122.08 | S7795 | ORY-1001 (RG-6016) 2HCl | 29.76 |
| S7591 | BRD4770 | 112.57 | S7953 | ETC-1002 | 28.80 |
| S8441 | LW6 | 79.06 | S7230 | UNC0642 | 27.80 |
| S1107 | Danusertib (PHA-739358) | 78.65 | S1327 | Ellagic acid | 26.84 |
| S7281 | JIB-04 | 77.78 | S1087 | Iniparib (BSI-201) | 25.47 |
| S7234 | IOX1 | 74.87 | S7062 | Pinometostat (EPZ5676) | 25.37 |
| S7572 | A-366 | 73.66 | S8353 | CPI-1205 | 23.56 |
| S1004 | Veliparib (ABT-888) | 69.48 | S7061 | GSK126 | 23.42 |
| S2554 | Daphnetin | 63.41 | S1149 | Gemcitabine HCl | 22.73 |
| S7595 | Santacruzamate A (CAY10683) | 61.95 | S8138 | Molidustat (BAY 85-3934) | 22.37 |
| S7796 | GSK2879552 2HCl | 60.00 | S4710 | Picolinamide | 20.98 |
| S8443 | MK-8617 | 59.16 | S1396 | Resveratrol | 20.78 |
| S2178 | AG-14361 | 58.99 | S7820 | EPZ020411 2HCl | 20.42 |
| S7300 | PJ34 HCl | 58.54 | S1484 | MC1568 | 20.04 |
| S7958 | Lificiguat(YC-1) | 57.56 | S8111 | GSK591 | 19.37 |
| S4715 | Benzamide | 54.15 | S8146 | Mitomycin C | 17.07 |
| S8038 | UPF 1069 | 52.68 | S1143 | AG-490 (Tyrphostin B42) | 17.01 |
| S8627 | OSS_128167 | 51.32 | S3147 | Entacapone | 16.50 |
| S3001 | Clevudine | 51.22 | S7128 | Tazemetostat (EPZ-6438) | 15.53 |
| S7656 | CPI-360 | 50.79 | S7292 | RG2833 (RGFP109) | 15.33 |
| S7979 | FG-2216 | 49.76 | S8479 | LLY-283 | 15.00 |
| S7619 | MI-3 (Menin-MLL Inhibitor) | 49.27 | S7476 | MG149 | 14.56 |
| S8113 | BI-9564 | 49.21 | S7438 | ME0328 | 13.41 |
| S7113 | Zebularine | 47.32 | S7004 | EPZ005687 | 11.58 |
| S7833 | OICR-9429 | 47.32 | S8460 | Salermide | 10.99 |
| S7680 | SP2509 | 46.83 | S7617 | Tasquinimod | 9.95 |
| S7593 | Splitomicin | 41.95 | S1509 | Norfloxacin | 9.74 |
| S7574 | GSK-LSD1 2HCl | 40.00 | S4294 | Procainamide HCl | 9.58 |
| S8245 | Thiomyristoyl | 39.37 | S8481 | SRT3025 HCl | 8.16 |
| S7832 | SGC707 | 37.17 | S8197 | APTSTAT3-9R | 7.83 |
| S7231 | GSK2801 | 37.07 | S4125 | Sodium Phenylbutyrate | 6.90 |
| S7296 | ML324 | 34.15 | S7353 | EPZ004777 | 6.34 |
| S8496 | EED226 | 32.98 | S8005 | SMI-4a | 5.76 |

| S1541 | Selisistat (EX 527) | 4.96 | S1950 | Metformin HCl | -12.40 |
| --- | --- | --- | --- | --- | --- |
| S7730 | NU1025 | 4.35 | S7088(1/4) | UNC1215 | -12.64 |
| S7616 | CPI-169 | 2.51 | S1168 | Valproic acid sodium salt (Sodium valproate) | -12.92 |
| S1995 | Procarbazine HCl | 1.01 | S7581 | GSK J1 | -13.04 |
| S2919 | IOX2 | 0.98 | S1373 | Daptomycin | -13.11 |
| S8664 | GSK3326595 (EPZ015938) | 0.53 | S1826 | Nedaplatin | -13.89 |
| S1215 | Carboplatin | 0.51 | S8340 | SGC2085 | -14.03 |
| S2686 | NVP-BSK805 2HCl | 0.21 | S8147 | MS049 | -14.35 |
| S7726 | BRD73954 | -1.30 | S3934 | Acetyl Resveratrol | -14.87 |
| S8287 | CPI-455 HCl | -1.30 | S1233 | 2-Methoxyestradiol (2-MeOE2) | -16.12 |
| S4170 | Coumarin | -1.53 | S7373 | UNC669 | -16.52 |
| S2298 | Fisetin | -2.06 | S2697 | A-769662 | -16.53 |
| S7612 | PX-478 2HCl | -2.17 | S7835 | I-BRD9 | -16.59 |
| S8419 | E7449 | -2.89 | S4246 | Tranylcypromine (2-PCPA) HCl | -17.24 |
| S7884 | AMI-1 | -3.04 | S7767 | SGI-1027 | -17.39 |
| S7238 | NVP-TNKS656 | -3.07 | S7237 | OG-L002 | -17.62 |
| S3770 | Sodium Aescinate | -4.47 | S7318 | HTH-01-015 | -17.62 |
| S8429 | PNU-74654 | -4.74 | S7265 | MM-102 | -18.54 |
| S7582 | Anacardic Acid | -4.78 | S2542 | Phenformin HCl | -18.82 |
| S7689 | BG45 | -5.65 | S7767 | AZ6102 | -20.21 |
| S7315 | PFI-3 | -5.75 | S8607 | JQ-EZ-05 (JQEZ5) | -20.31 |
| S7618 | MI-2 (Menin-MLL Inhibitor) | -5.85 | S8370 | BGP-15 2HCl | -20.52 |
| S4771 | β-thujaplicin | -6.51 | S7294 | PFI-2 HCl | -21.07 |
| S8601 | CP2 | -6.82 | S2789 | Tofacitinib (CP-690550,Tasocitinib) | -21.46 |
| S7165 | UNC1999 | -7.28 | S2804 | Sirtinol | -21.46 |
| S5001 | Tofacitinib (CP-690550) Citrate | -7.28 | S1648 | Cytarabine | -21.72 |
| S2759 | CUDC-907 | -7.32 | S7681 | OF-1 | -22.44 |
| S1384 | Mizoribine | -7.58 | S1802 | AICAR (Acadesine) | -23.14 |
| S7239 | G007-LK | -8.05 | S8359 | UNC3866 | -23.66 |
| S7577 | AGK2 | -8.26 | S8209 | HLCL-61 HCL | -24.39 |
| S8323 | ITSA-1 (ITSA1) | -8.59 | S1007 | Roxadustat (FG-4592) | -24.53 |
| S7748 | EPZ015666(GSK3235025) | -8.70 | S4900 | Tenovin-6 | -26.44 |
| S8180 | PF-CBP1 HCl | -8.70 | S1703 | Divalproex Sodium | -27.45 |
| S8112 | MS023 | -9.76 | S4735 | Salvianolic acid B | -28.05 |
| S8249 | HPI-4 (Ciliobrevin A) | -10.43 | S7804 | GSK503 | -28.26 |
| S7845 | SirReal2 | -10.87 | S1132 | INO-1001 (3-Aminobenzamide) | -28.72 |
| S8171 | Daprodustat (GSK1278863) | -11.30 | S7229 | RGFP966 | -29.50 |
| S7070 | GSK J4 HCl | -11.88 | S1999 | Sodium butyrate | -30.30 |
| S7611 | EI1 | -12.17 | S7946 | KC7F2 | -30.58 |

| S7541 | Decernotinib (VX-509) | -30.65 | S7256 | SGC-CBP30 | -57.47 |
| --- | --- | --- | --- | --- | --- |
| S1899 | Nicotinamide  (Vitamin B3) | -30.99 | S7278 | HPOB | -59.00 |
| S8265 | GSK6853 | -31.74 | S2902 | S-Ruxolitinib (INCB018424) | -60.15 |
| S1200 | Decitabine | -31.76 | S1147 | Barasertib (AZD1152-HQPA) | -61.93 |
| S7555 | 4SC-202 | -33.33 | S8502 | TMP195 | -63.46 |
| S8056 | Lomeguatrib | -33.48 | S8495 | WT161 | -63.68 |
| S2818 | Tacedinaline (CI994) | -34.33 | S7605 | Filgotinib (GLPG0634) | -64.43 |
| S8363 | NMS-P118 | -34.66 | S1103 | ZM 447439 | -64.59 |
| S7065 | MK-8745 | -34.77 | S7641 | Remodelin | -64.61 |
| S2770 | MK-5108 (VX-689) | -35.25 | S2851 | Baricitinib (LY3009104, INCB028050) | -65.13 |
| S1129 | SRT1720 HCl | -36.16 | S4589 | Amodiaquine dihydrochloride dihydrate | -65.52 |
| S7317 | WZ4003 | -36.78 | S7570 | UNC0379 | -65.56 |
| S7295 | Apabetalone (RVX-208) | -36.78 | S7816 | MI-463 | -66.22 |
| S8270 | SRT2183 | -36.96 | S8006 | BIX 01294 | -66.28 |
| S7419 | Blasticidin S HCl | -37.12 | S7815 | MI-136 | -66.61 |
| S7324 | TMP269 | -37.55 | S2867 | WHI-P154 | -66.67 |
| S7906 | PFI-4 | -38.70 | S7189 | I-BET-762 | -67.05 |
| S1100 | MLN8054 | -38.83 | S1053 | Entinostat (MS-275) | -67.22 |
| S1060 | Olaparib (AZD2281, Ku-0059436) | -40.10 | S7306 | Dorsomorphin (Compound C) 2HCl | -68.69 |
| S2821 | RG108 | -40.23 | S8004 | ZM 39923 HCl | -68.70 |
| S2407 | Curcumol | -40.39 | S1048 | Tozasertib (VX-680, MK-0457) | -68.75 |
| S8000 | Tenovin-1 | -40.87 | S2791 | Sotrastaurin | -69.35 |
| S7625 | Niraparib (MK-4827) tosylate | -42.17 | S1848 | Curcumin | -69.48 |
| S7119 | Go6976 | -44.39 | S8049 | Tubastatin | -69.53 |
| S8494 | PF-06726304 | -45.00 | S8179 | BI-7273 | -69.61 |
| S7079 | SGC 0946 | -45.21 | S1782 | Azacitidine | -69.92 |
| S2250 | (-)-Epigallocatechin Gallate | -45.32 | S2744 | CCT137690 | -70.00 |
| S2740 | GSK1070916 | -46.70 | S8567 | Tucidinostat (Chidamide) | -70.26 |
| S2796 | WP1066 | -46.74 | S7152 | C646 | -70.88 |
| S8071 | UNC0638 | -46.96 | S2018 | ENMD-2076 L-(+)-Tartaric acid | -71.49 |
| S8096 | Mirin | -47.23 | S1774 | Thioguanine | -71.57 |
| S7843 | BI-847325 | -47.39 | S7041 | CX-6258 HCl | -72.39 |
| S2179 | Gandotinib (LY2784544) | -47.93 | S2627 | Tubastatin A HCl | -72.84 |
| S7104(1/4) | AZD1208 | -48.28 | S1393 | Pirarubicin | -73.76 |
| S1573 | Fasudil (HA-1077) HCl | -49.80 | S2911 | Go 6983 | -73.95 |
| S2197 | A-966492 | -50.21 | S1181 | ENMD-2076 | -73.97 |
| S7305 | MS436 | -51.72 | S1133 | Alisertib (MLN8237) | -74.08 |
| S7817 | MI-503 | -55.22 | S1216 | PFI-1 (PF-6405761) | -74.49 |

| S2012 | PCI-34051 | -74.78 | S2341 | (-)-Parthenolide | -93.60 |
| --- | --- | --- | --- | --- | --- |
| S7575 | LLY-507 | -75.96 | S2198 | SGI-1776 free base | -94.00 |
| S7616 | BMS-911543 | -76.43 | S2692 | TG101209 | -94.01 |
| S1422 | Droxinostat | -78.04 | S8195 | Oclacitinib maleate | -94.14 |
| S7473 | Nexturastat A | -78.16 | S2693 | Resminostat | -94.63 |
| S7983 | A-196 | -79.43 | S7569 | LMK-235 | -95.02 |
| S1098 | Rucaparib (AG-014699,PF-01367338) phosphate | -80.59 | S1122 | Mocetinostat (MGCD0103) | -95.13 |
| S1030 | Panobinostat (LBH589) | -80.99 | S7620 | GSK1324726A (I-BET726) | -95.57 |
| S7110 | (+)-JQ1 | -81.61 | S2244 | AR-42 | -96.45 |
| S8190 | CPI-637 | -81.78 | S8043 | Scriptaid | -96.83 |
| S1095 | Dacinostat (LAQ824) | -82.02 | S2718 | TAK-901 | -97.18 |
| S2779 | M344 | -85.82 | S2736 | Fedratinib (SAR302503, TG101348) | -97.21 |
| S7036 | XL019 | -85.82 | S1134 | AT9283 | -97.37 |
| S7029 | AZD2461 | -86.21 | S1515 | Pracinostat (SB939) | -97.38 |
| S8648 | ACY-738 | -86.84 | S2170 | Givinostat (ITF2357) | -97.57 |
| S1451 | Aurora A Inhibitor I | -87.69 | S1194 | CUDC-101 | -97.57 |
| S7360 | OTX015 | -87.95 | S2219 | Momelotinib (CYT387) | -97.72 |
| S1171 | CYC116 | -88.43 | S2162 | AZD1480 | -98.22 |
| S1378 | Ruxolitinib (INCB018424) | -89.88 | S8001 | Ricolinostat (ACY-1215) | -98.48 |
| S7304 | CPI-203 | -90.17 | S1085 | Belinostat (PXD101) | -98.54 |
| S7650 | Peficitinib (ASP015K, JNJ-54781532) | -90.47 | S1096 | Quisinostat (JNJ-26481585) 2HCl | -98.67 |
| S8400 | Mivebresib(ABBV-075) | -90.79 | S8057 | Pacritinib (SB1518) | -98.78 |
| S1154 | SNS-314 | -91.01 | S7588 | Reversine | -98.85 |
| S8464 | Citarinostat (ACY-241) | -91.41 | S7259 | FLLL32 | -98.98 |
| S1529 | Hesperadin | -91.53 | S7650 | Bromosporine | -99.00 |
| S8344 | AZD5153 | -92.04 | S7634 | Cerdulatinib (PRT062070, PRT2070) | -99.43 |
| S1249 | JNJ-7706621 | -92.13 | S8068 | Chaetocin | -99.52 |
| S1047 | Vorinostat (SAHA, MK0683) | -92.15 | S2214 | AZ 960 | -99.61 |
| S1090 | Abexinostat (PCI-24781) | -92.56 | S7596 | CAY10603 | -99.64 |
| S2158 | KW-2449 | -92.64 | S2719 | AMG-900 | -99.66 |
| S2806 | CEP-33779 | -93.27 |  |  |  |
